# Supplementary material for: A compliant metastructure design with reconfigurability up to six degrees of freedom
Source: Nat Commun. 2025 Jan 16;16:719. doi: 10.1038/s41467-024-55591-2 (PMC11739503; doi:10.1038/s41467-024-55591-2)
Supplement: Supplementary file 1 — Supplementary Information [file 41467_2024_55591_MOESM1_ESM.pdf]

Supplementary Information
A Compliant Metastructure Design with Reconfigurability up to Six Degrees of Freedom

Humphrey Yang[1], Dinesh K. Patel[1,2], Tate Johnson[1,3], Ke Zhong[1,4], Gina Olson[5], Carmel Majidi[2], Mohammad F. Islam[4], Teng Zhang[6,7*], Lining Yao[1,8*]

Affiliations

[1]Morphing Matter Lab, Human-Computer Interaction Institute, Carnegie Mellon University, Pittsburgh, PA, United States.
[2]Department of Mechanical Engineering, Carnegie Mellon University, Pittsburgh, PA, United States.
[3]School of Design, Carnegie Mellon University, Pittsburgh, PA, United States.
[4]Materials Science and Engineering, Carnegie Mellon University, Pittsburgh, PA, United States.
[5]Mechanical and Industrial Engineering, University of Massachusetts Amherst, MA, United States.
[6]Department of Mechanical and Aerospace Engineering, Syracuse University, Syracuse, NY, United States.
[7]BioInspired Syracuse, Syracuse University, Syracuse, NY, United States.
[8]Mechanical Engineering, University of California, Berkeley, Berkeley, CA, United States.
*Correspondence to: tzhang48@syr.edu, liningy@berkeley.edu.

This PDF file includes

Supplementary Note 1: Materials, Fabrication, and Control
Supplementary Note 2. Rational Design Algorithm
Supplementary Note 3: Stiffness Model and Finite Element Simulation
Supplementary Note 4: 6-DOF Device Design
Supplementary Note 5. Wearable Haptic Device Design and Iteration
Supplementary Note 6: Material Characterization and Mechanical Experiment of Devices

Supplementary Note 1: Materials, Fabrication, and Control

1.1 Fabrication of Stiffness-Changing Flexural Rods.

The stiffness-changing flexural rods are prepared by casting in customized jigs (Fig. S1). The jig is machined from a 6061 aluminum stock (McMaster-Carr) and has hemicylindrical grooves, alignment features, and securing screws and nuts for lining up heating wires (34-gauge 316L stainless steel wire, Master Wire Supply) at the center of the rods. To cast the rods, silicone tubes (2 mm ID * 3 mm OD or 1.5 mm ID * 3 mm OD, uxcell) are cut into desired lengths, placed on the grooves, and taped (Kapton masking tape, McMaster-Carr) to the jig to allow for threading heating wires through their center. The alignment features on both ends of the grooves are used to ensure the heating wires are positioned at the center of the silicone tubes. Once the heating wires are tightened and straightened, the screws and nuts on the far ends are used to secure them throughout the fabrication process.

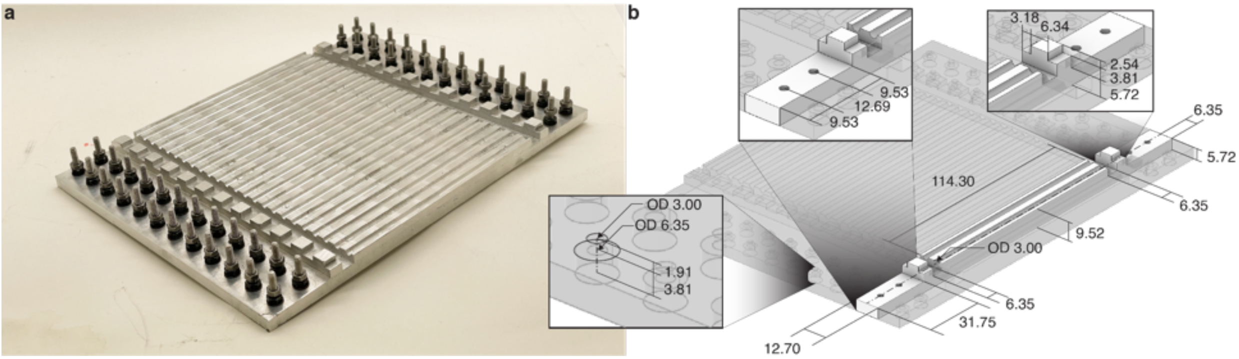

Figure S1. Stiffness-changing flexural rod casting jig design. a*, The machined jig prior to casting flexural rods.* ***b****, Schematic diagram of a single unit on the jig. Unit, mm.*

To prepare the casting resin solution, Bisphenol A Epichlorohydrin-based epoxy resin (Hexion EPON resin 828, monomer) and the curing agent (Epikure 3380, cross-linker) are combined at a weight ratio of 10:4, respectively. The resin is mixed and degassed for three minutes each using a planetary centrifugal mixer (Thinky AR-100) to derive a uniform, bubbleless solution. The mixed resin is then loaded into a syringe with an 18- or 22-gauge dispensing tip and injected into the silicone tubes on the aluminum jig (Fig. S2). Once cast, the resin is left to gel at room temperature (25°C) for twenty-four hours, followed by thermal curing at 100°C in an oven (725F, Thermo Fisher Scientific) for five hours to crosslink fully. Cured resin rods, along with the jig, are then removed from the oven and left to cool down to room temperature. The rods sheathed in silicone tubes are then released from the aluminum jig by removing the tape and loosening the nuts that secured the heating wires. Finally, the rods are unsheathed by slicing and peeling off the silicone tubes.

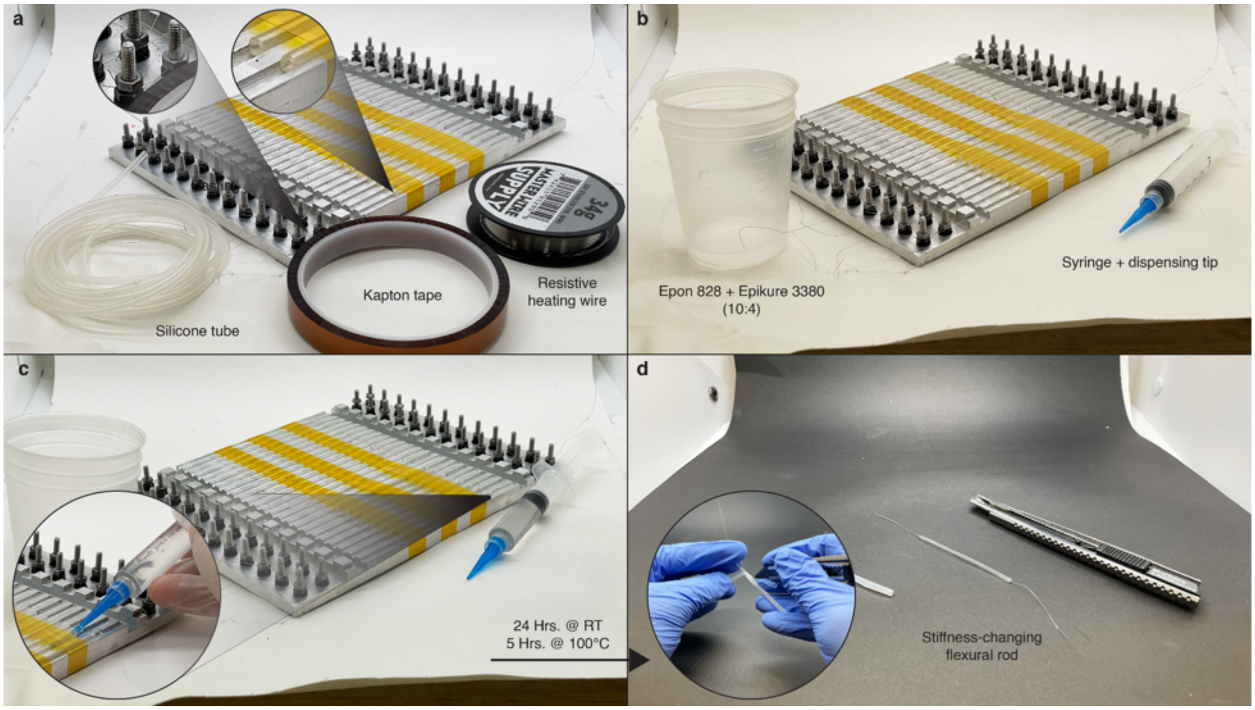

Figure S2. The fabrication process of stiffness-changing flexural rods. a*, Add tubes and secure resistive heating wires to the jig. Inset images show the wire and tube alignment and the screws securing the heating wires on both ends.* ***b****, Mixing epoxy solution for injection into the molds.* ***c****, injecting epoxy into the tubes. Inset image, injecting the solution from the end of tubes using a 5 ml syringe and a 22-gauge industrial dispensing tip.* ***d****, Releasing the stiffness-changing rods from the tubes. The inset image shows a knife slicing the tube open, which was then peeled off from the rod.*

1.2 Fabrication of Rigid Stages, Testing Jigs, and Passive Flexures.

The rigid stages and testing jigs used and demonstrated in this work are designed in Rhinoceros 3D version 7 and exported as STL files for fabrication. The parts are made with either a fuse-deposition modeling (FDM) (Ultimaker S5) or a stereolithography (SLS) 3D printer (Formlabs 3B).

White polylactic acid (PLA) filaments and 0.4 mm extruder nozzles are purchased from Ultimaker and are used to fabricate the 6DOF devices' rigid stages and the fixture for mechanical tests. In the printing processor (Ultimaker Cura), the parts are sliced with default settings at a layered height of 0.1 mm and a 60% gyroid infill for increased mechanical strength. Both adhesion plates and structural supports are enabled to ensure print quality. The passive flexures used in the wearable device are also printed with PLA but with a 0.25 mm nozzle for higher resolution. The flexures are sliced at a 0.1 mm layer height with a 100% infill rate to produce solid objects.

All other parts are made with the SLS printer using the Formlabs White resin V4 with default settings with a layer height of 0.1 mm. SLS-printed parts are washed with isopropyl alcohol to remove resin residue and flood-UV cured at 60°C for 60 minutes using Formlabs' FormCure post-curing machine.

1.3 Assembling Stages with Flexural Rods.

The rigid stages are designed with circular through holes for housing the rods (Fig. S3). The rods are inserted into their designated holes to assemble them to the stages, followed by applying cyanoacrylate adhesives (Scotch-Weld, 3M) at the interface. The adhesive is then left to dry at room temperature for twenty-four hours. Finally, the heating wires are connected to conductive wires by ferrules (Copper rivets 0.4 mm, Voltera) 3 mm away from the end of the rods where the heating wires are exposed.

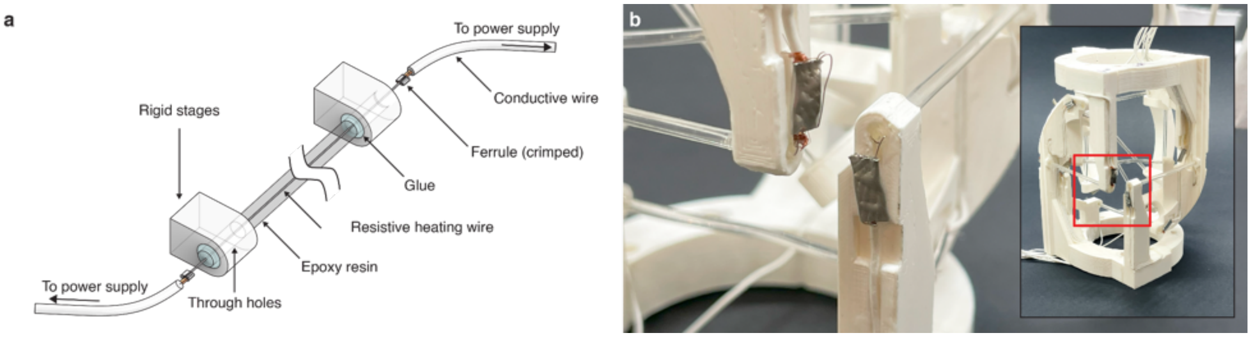

***Figure S3. Structure assembly and connection. a**, A schematic diagram of the connection between the flexures and rigid stages, as well as electrical connections. **b**, A picture of the assembled 6-DOF device showing the connections.*

1.4 Heating Control

To heat a stiffness-changing flexural rod above its glass transition temperature, we connect the two ends of the conductive wires to a power supply (SPS 3010, Kungber) and provide a current of 0.4 A to the resistive heating wire (rated resistance: 3.68×10^{-2} Ω/mm), which corresponds to 5.88×10^{-3} watts/mm. The voltage is adjusted depending on the length of a rod and scales linearly with its length. It takes 31.45 ± 2.58 seconds to heat a rod from ambient condition (25°C) to above their glass-transition temperature (T_g, 54°C), yet in our experiments (see following sections), we allow the rods to heat for three minutes to reach quasi-thermal equilibrium. On the other hand, to cool down a rod below its T_g, we cut off the current and allow it to passively dissipate heat in ambient conditions. It takes 67.90 ± 4.95 seconds for a rod to cool from its glass transition temperature to the ambient temperature and allow the rods to cool for three minutes in our experiments without extraneous loads. Any external load may cause the rod to retain in a deformed state as it cools down, which may unintentionally alter the device's kinematics freedoms.

Multiple rods can be simultaneously heated by serially connecting their heating wires end-to-end (Fig. S4). We manually connect and disconnect the wires to alter the circuit and switch rods between their cold and hot states to reconfigure kinematic modes. While not implemented in this work, future iterations may consider using relays and transistors to afford digital control and reconfiguration of the devices.

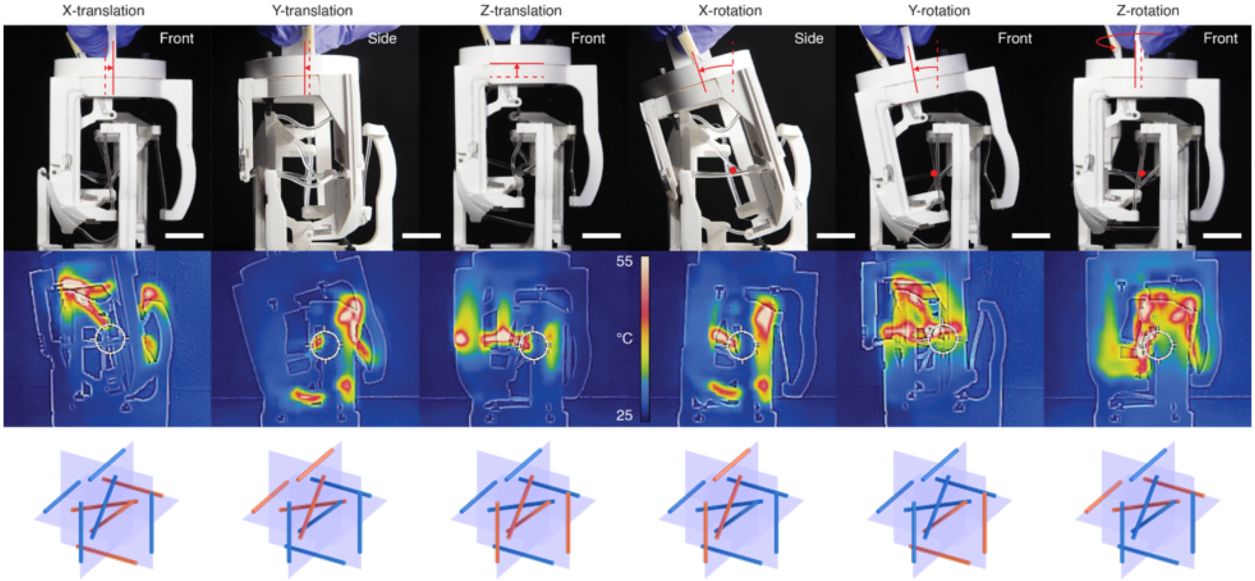

Figure S4. Thermal images of the 6-DOF device under different mode configurations. *Top row, picture of the device; middle row, thermal images; bottom row, rod configuration (matching Fig. 3c). Blue rods are configured cold while rods in orange/red are heated.*

The epoxy rods' mechanical property is a function of temperature (see also Section 6.1). The environmental temperature mainly affects the rods' stiffness under the unheated state. The higher the ambient temperature, the softer the epoxy rod becomes in the unheated state. Consequently, a rod would exhibit a smaller stiffness change between the two states. The epoxy's tan delta remains more or less constant between 30°C to 40°C, and the moduli are still magnitudes higher than that in the heated state and sufficient to lock a DOF. Literature has shown that epoxy's glass transition temperature[1] and stiffness[2] can be tuned by altering the crosslinker ratio: the higher the crosslinker concentration, the higher the Tg and stiffness. When designing devices for unusually hot environments, using a higher crosslinker ratio may help to create a desired stiffness change and avoid undesired softening due to temperature fluctuations.

On the other hand, heating and cooling time may also be affected by the ambient temperature. At 25°C, a rod takes 31.45 ± 2.58 seconds to heat up to 54°C and 67.90 ± 4.95 seconds to cool down. At 30°C, the heating and cooling time are reduced to 29.12 ± 2.39 and 40.72 ± 3.01 seconds, respectively. At 35°C, the heating and cooling time are further reduced to 25.62 ± 2.18 and 26.31 ± 2.06 seconds, respectively.

1.5 Notes on Material Selection and Safety

In this work, we use epoxy to make stiffness-changing flexures for its availability, customizability, and ease of control. In particular, epoxy is a common material for making stiffness-changing components in robotic systems[3]. Their glass transition temperature could be tuned by altering the ratio between the crosslinker and monomer during its synthesis[1]. The material could also be conveniently made into different shapes[1] by casting, printing, or laser cutting, thus providing high customizability.

The monomer-to-crosslinker ratio was selected to produce a suitable glass transition temperature. In particular, a higher monomer crosslinker fraction will produce flexures with a higher T_g as the polymer chains require more energy to recoil. In contrast, a lower T_g can be obtained by reducing the crosslinker fraction. A T_g of 54°C was chosen because it is relatively close to the body temperature but sufficiently high to be insensitive to ambient heat fluctuations (e.g., body heat, warm water, in a wearable context). Moreover, a crosslinker that leads to a lower T_g would also compromise the flexure's mechanical strength due to reduced crosslinking density[1]. Regarding safety, we note that the literature[4] had reported that the skin's exposure tolerance to heat is a function of the temperature, and the safe time against a 54°C heat source is 14.04 seconds without incurring strong thermal injury[4]. Still, extended exposure may lead to first or second-degree burns, and insulation or protection is required[5]. In this work, we assure safety by placing the flexure rods away from the skin to avoid collision and covering the skin with fabrics.

Supplementary Note 2. Rational Design Algorithm

This supplementary note describes the rational design using the wrist joint shown in Figure 2 as an example. Section 2.1. covers the background of representing and designing compliant mechanisms joints with screw algebra. The later sections describe the algorithm and information relevant to multimodal kinematics design.

2.1 Background: Analysis and Design of Parallel Flexure Compliant Mechanisms Using Screw Algebra

This section summarizes the algebraic representation and modeling required to understand the reconfigurable kinematics design algorithm. The mathematical foundation has been introduced by Johnathan et al.[6–8] We refer readers to the literature for further details.

Representing motional freedoms and constraints as screw vectors

Compliant mechanisms' kinematics design through the screw theory has been demonstrated by the literature[6,7,9–13]. In the 3D space, the instantaneous motion $\widehat{\mathbf{T}}$ of a body can be described using a 6×1 screw vector:

$$\widehat{\mathbf{T}} = \omega[\widehat{\mathbf{n}} \quad (\widehat{\mathbf{c}} \times \widehat{\mathbf{n}}) + p\widehat{\mathbf{n}}]^{\mathrm{T}} \qquad \text{(eq. 1)}$$

Where $\widehat{\mathbf{T}}$ is also called a twist vector, ω is the motion's angular velocity, and p is the pitch of the motion (i.e., translation along the screw axis per revolution). $\widehat{\mathbf{n}}$ and $\widehat{\mathbf{c}}$ are 3×1 vectors that describe the screw axis and a reference point on the axis, respectively. Specifically, $\widehat{\mathbf{n}}$ should be a unit vector pointing along the motional axis, and $\widehat{\mathbf{c}}$ is a vector that points from the spatial origin to any point along the axis. For pure rotational motions, p is zero; for pure translational motions, the motion has an infinite pitch, leading to the following form after normalization (i.e., divide by infinity[6]:

$$\widehat{\mathbf{T}} = \upsilon[\widehat{\mathbf{0}} \quad \widehat{\mathbf{n}}]^{\mathrm{T}} \qquad \text{(eq. 2)}$$

Where υ is the translational velocity and $\widehat{\mathbf{0}}$ is a 3×1 zero vector.

Similar to motional freedoms, constraints imposed by flexure elements can also be described using screw vectors. For a linear rod flexure, its one degree of constraint load $\widehat{W}$ can be described as

$$\widehat{\mathbf{W}} = f[\widehat{\mathbf{n}} \quad (\widehat{\mathbf{r}} \times \widehat{\mathbf{n}}) + q\widehat{\mathbf{n}}]^{\mathrm{T}} \qquad \text{(eq. 3)}$$

Where $\widehat{\mathbf{W}}$ is also called a wrench vector, f is the constraining force magnitude, and the reference point $\widehat{\mathbf{c}}$ and screw pitch p in (eq. 1) are replaced with new notations $\widehat{\mathbf{r}}$ and q, respectively. Figure S5 provides a summary of these notations. In this work, we also refer to the first half of a screw vector as the directional component and the latter as the positional component.

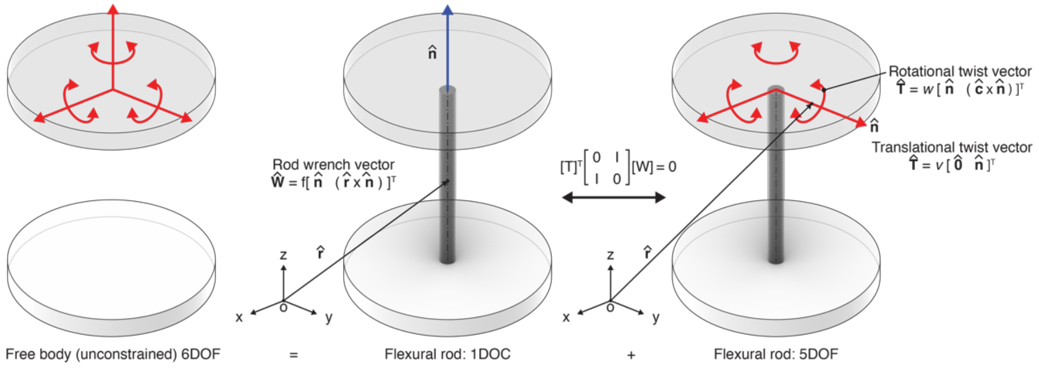

Figure S5. Screw algebraic representation of a single flexural rod.

Linear space representation of compliant mechanism joint freedoms and constraints

A rigid body in 3D space may have up to six independent degrees of freedom (i.e., translation and rotation along the principal axes) at the same time. For a compliant mechanical system consisting of two rigid stages connected by parallel flexures, the free end may have $n \in [0, 6]$ DOF with respect to the fixed end, depending on the flexure layout. In particular, the free end can move in any combination of the enabled DOF, and the collection of permissible motions can be represented as a $6 \times n$ linear subspace [T] spanned by the independent DOF:

$$\widehat{\mathbf{J}_\mathbf{f}} = [\mathrm{T}]\hat{\mathbf{x}},\ \hat{\mathbf{x}} = [x_1\ \dots\ x_n]^{\mathrm{T}} \qquad \text{(eq. 4)}$$

Where $\widehat{\mathbf{J}_\mathbf{f}}$ is the joint's permissible motions, $\hat{\mathbf{x}}$ is a $n \times 1$ velocity vector for each independent DOF, and [T] is the freedom space afforded by the joint. Specifically, [T] is a matrix consisting of n independent unit twist vectors, each describing a DOF afforded by the joint, and $\hat{\mathbf{x}}$ is the parameter for generating any motion afforded by the design.

When designing compliant mechanisms consisting of parallel flexures, given a set of twist vectors representing the desired motional freedoms between the stages, the targeted freedom space can be computed by appending the twist vectors into a $6 \times n'$ ($n' \geq n$) matrix [T′], finding its echelon form, and eliminating zero rows. Note that the twist vectors in [T′] should describe the free stage's motion with respect to the fixed stage. The rank of the resulting matrix equates to the number of independent DOF. Alternatively, the freedom space can also be found by computing the kernel of its kernel, i.e.,

$$[\mathrm{T}] = \mathcal{N}(\mathcal{N}([\mathrm{T}'])) \qquad \text{(eq. 5)}$$

The compliant flexures between two rigid stages can also be represented as a constraint space [W] that is derived using the same method as [T], but the twist vectors are replaced by wrench vectors representing the flexural elements. The constraint space's rank m equates to the compliant

mechanism's independent degrees of constraints. Thus, a joint's permissible constraint forces $\widehat{\mathbf{J_c}}$ can be represented with

$$\widehat{\mathbf{J_c}} = [\mathrm{W}]\hat{\mathbf{y}},\ \hat{\mathbf{y}} = [y_1\ \cdots\ y_m]^{\mathrm{T}} \qquad \text{(eq. 6)}$$

Where $\hat{\mathbf{y}}$ represents the constraining force magnitude as a $m \times 1$ vector.

There exists a mapping between a compliant mechanism's freedom and constraint spaces [T] and [W]:

$$[\mathrm{T}]^{\mathrm{T}} \begin{bmatrix} 0 & \mathrm{I} \\ \mathrm{I} & 0 \end{bmatrix} [\mathrm{W}] = [0] \qquad \text{(eq. 7)}$$

Where the freedom and constraint spaces are correlated by a swap operator[14] consisting of I and 0 as 3×3 identity and zero submatrices, respectively. Thus, given any of [T] or [W], the other can be found by solving eq. 7. It is worth noting that following Maxwell's equation, a compliant mechanism's number of DOF and DOC sums to six (Fig. S5), thus:

$$6 = n + m \qquad \text{(eq. 8)}$$

Note that it is assumed that the stages can assume any shape as long as they are sufficiently rigid and does not provide additional DOF to the compliant mechanism. Under this assumption, the kinematics of a compliant mechanism is solely determined by the flexure layout, and the stages have their shapes adapted for any targeted function.

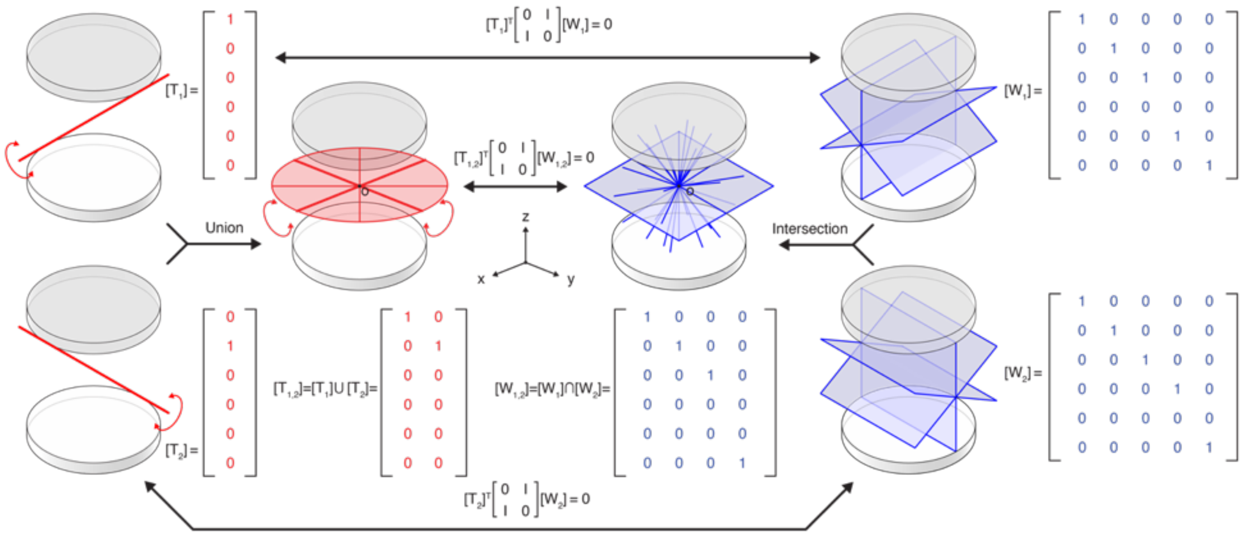

Figure S6. Using screw linear subspaces to represent a compliant joint kinematics and flexure design. *The screw subspace union and intersection are also shown in this image, using the wrist joint's kinematic freedoms as an example.*

Visualization of freedom and constraint spaces

A freedom space describes the direction and position of a joint's permissible motions. Similarly, the constraint space describes the direction and position of constraint lines (i.e., flexural rod axis). While screw vectors are inherently six-dimensional, they can be broken down into their constituent components for visualization in the 3D space. In particular, the axis direction ($\hat{\mathbf{n}}$) and reference point ($\hat{\mathbf{c}}$, $\hat{\mathbf{r}}$) components can be used to find the placement and orientation of the motional or constraint axis. For twist vectors, if the L2 norm of the directional component equals zero (i.e., $||\hat{\mathbf{n}}||_2 = 0$), then the motion is a pure translation, and the motional axis can lie anywhere in space. Conversely, if $||\hat{\mathbf{n}}||_2 \neq 0$, the motion is a pure rotation if $p = 0$ and a screw motion otherwise. A placement is only valid for wrench vectors when $||\hat{\mathbf{n}}||_2 \neq 0$, since a zero-vector directional component implies a flexural rod without direction.

On the other hand, freedom and constraint spaces [T] and [W] can also be interpreted using an identical method. In this case, the spaces can be regarded as implicit geometries with the velocity vector $\hat{\mathbf{x}}$ in eq. 4 or force magnitude vector $\hat{\mathbf{y}}$ in eq. 6 as the parameters. The independent vectors in [T] and [W] can be linearly combined to produce motional or constraint subspaces (i.e., point, line, plane, or volume), which in turn describes the shape spanned by permissible motional axes for [T] and flexure placements for [W] in the 3D space. Hopkins et al.[6] have provided a comprehensive library of different [T] and [W] combinations. We refer readers to the literature for more details.

Constraint space validity

A constraint space is only valid when it satisfies two requirements. First, the space must have at least one non-zero directional component in its constituent vectors, or else the constraint space cannot produce a valid placement for flexural rods (i.e., axis-less rods). Second, the space must be able to produce wrench vectors that have $q = 0$.

Constraint space completion

When designing a compliant mechanism through the freedom and constraint topology[6,7] (FACT) method, the flexures within the system should be placed to exactly constrain the device's DOF. That is, the space $[W_{flx}]$ spanned by the flexures' wrench vectors should match that required by the targeted constraint space $[W_{tar}]$ without additional independent wrench vectors in $[W_{flx}]$. In this case, the following relation should be true:

$$\mathcal{N}([W_{tar}])[W_{flx}] = [0] \quad \text{(eq. 9)}$$

If eq. 9 evaluates to false, the design is over-constrained and may cause the design to have fewer or mismatching degrees of freedom than intended. On the other hand, under-constraining design can also be identified by checking the rank of $[W_{flx}]$ against $[W_{tar}]$. For an exactly constrained design, the following relation should be true:

$$\text{Rank}([W_{tar}]) - \text{Rank}([W_{flx}]) = 0 \quad \text{(eq. 10)}$$

Alternatively, eq. 10 can be replaced by the following condition:

$$\mathcal{N}([W_{flx}])[W_{tar}] = [0] \quad \text{(eq. 11)}$$

Eq.9 and eq. 11 are both true if and only if $[W_{flx}]$ and $[W_{tar}]$ have the same ranks and are equivalent (i.e., spanning the same linear subspace). The mechanism is underconstrained if $[W_{flx}]$ has a lower rank than $[W_{tar}]$ and over-constrained if $[W_{flx}]$ has a higher rank than $[W_{tar}]$. If a design is under-constrained, more flexural rods should be added until both eq.9 and eq.11 evaluate to true; if the design is instead over-constrained, the over-constraining flexure(s) should be removed. Over-constraining rods can be identified by checking eq. 9, but with individual flexures' wrench vectors replacing $[W_{flx}]$. The minimum number of flexural rods required to complete a constraint space is the same as the constraint space's rank. However, more rods can be added to achieve targeted device performance (e.g., structural or stiffness demands) so long as they are added within the constraint space. We use the notation $[W_a] \leftrightarrow [W_b]$ when $[W_a]$ is completed by $[W_b]$.

In this work, we manually and iteratively added flexure rods and checked eq. 9 and eq. 11 to make sure a target constraint space $[W_{tar}]$ is exactly constrained and satisfied. When generating a flexure layout $[W_{flx}]$, we linearly combine the basis vectors in $[W_{tar}]$ to create flexure wrench screws, which is then decomposed into flexure placement information (i.e., wrench axis as the orientation and a reference point along the axis's extended line). During the process, if the $[W_{flx}]$ was

underconstrained with respect to $[W_{tar}]$, we identify missing parts (i.e., span vectors) and use it in generating the next flexure placement. Conversely, if a flexure was over-constraining, we modify its placement to remove the over-constraining parts of its wrench vector.

2.2 Freedom and Constraint Space Boolean Operation.

Three operations are frequently used when designing a reconfigurable kinematic device - subspace union, intersection, and relative complement (also termed difference). The union (Fig. S6) of two freedom spaces $[T_a]$ and $[T_b]$ is defined as

$$[T_a] \cup [T_b] = \mathcal{N}(\mathcal{N}([T_{ab}])) \quad \text{(eq. 12)}$$

The two consecutive kernel operators are used to make sure the union subspace is spanned by linearly independent vectors, and $[T_{ab}]$ is the concatenation of the two linear systems along the first dimension. A union operator is useful in finding the summed freedom or constraint space between multiple kinematic modes. On the other hand, the intersection operator (Fig. S6) allows us to find the freedom or constraint subspace shared by different kinematic modes:

$$[T_a] \cap [T_b] = \mathcal{N}\left(\mathcal{N}([T_a])^T \cup \mathcal{N}([T_b])^T\right)^T \quad \text{(eq. 13)}$$

Finally, the difference operator (Fig. S7) allows us to find the subspace used by one space but not the other(s). The difference between the two freedom subspaces $[T_a]$ and $[T_b]$ (from $[T_b]$ to $[T_a]$) is defined as

$$[T_a] \backslash [T_b] = \mathcal{N}\left(\mathcal{N}([T_a])^T \cup [T_b]\right)^T \quad \text{(eq. 14)}$$

Additionally, when comparing two subspaces, one space may be fully spanned by the other. In that case, we define

$$[T_a] \subseteq [T_b] \Leftrightarrow \mathcal{N}([T_a])[T_b] = [0] \quad \text{(eq. 15)}$$

When $[T_a]$ is included by $[T_b]$ (i.e., $[T_a]$ is a subset of $[T_b]$). The same notation is also used for screw vectors. Like freedom spaces, constraint spaces can also be computed with the same schema to find their intersections, unions, and differences, but [T] is replaced by [W].

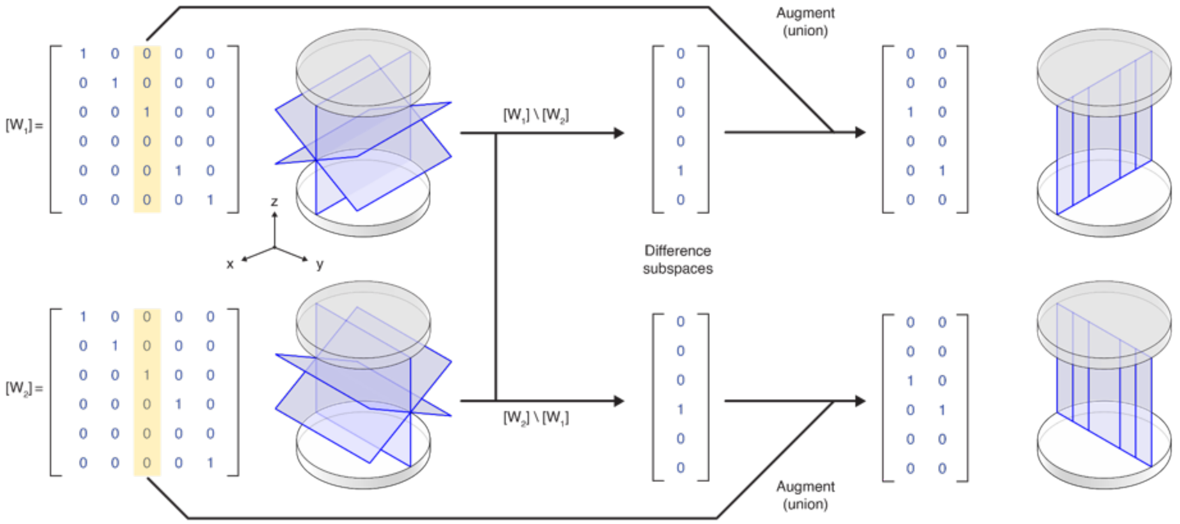

Figure S7. Constraint subspace Boolean differences. *This example shows the derivation of the wrist joint (Fig. 2) constraint subspaces. The operation produced unviable constraint subspaces, which were then supplemented to make them viable.*

2.3 Algorithm for Designing Reconfigurable Devices

The goal of this algorithm is to design a kinematic device that affords distinct kinematic modes (i.e., DOF) by selectively softening/stiffening flexural rods within the device (Fig. S8). The algorithm takes k numbers of kinematic modes $\mathrm{T} = \{[\mathrm{T}_1'], \cdots, [\mathrm{T}_k']\}$ as input, each described by a set of twist vectors defining their motional freedoms. Next, given the targeted modes, the algorithm finds the allowed placements of non-reconfigurable and stiffness-changing flexural rods. Step 2 finds the shared constraint spaces between kinematic modes, where rods are not required to be actively stiffness-changing, whereas steps 3 through 5 identify and create the minimal actively stiffness-changing constraint topology to achieve kinematic modal reconfigurations. Step 6 further allows users to add redundant flexures to achieve targeted kinesthetic performances.

We note that steps 2 through 5 are extensions made from the FACT method[6,7] to handle modal reconfigurations, which have also been discussed in a previous work[15]. However, previous work only presented the high-level design concept without rigorous and detailed formulation for implementation. More importantly, steps 4 through 6 incorporated newly introduced rules for augmenting constraint spaces to create more placement options, which are critical to wearable device design where available flexure placements are often confined by the user's body. Therefore, introducing constrain space augmentation rules navigates a larger design space and provides more freedom for flexural rod placements while achieving the targeted kinematic reconfigurations. Finally, the previous work used tensioning cables for kinematic reconfiguration, which could be subjected to tensile and compressive loads in the relaxed (unlocked state) without failure. Yet, the

actively stiffness-changing flexures used in this work could only be compressed, not extended, and section 2.7 further introduces an orientation check to make sure the active flexures experience a legal (compressive) load in their unlocked state.

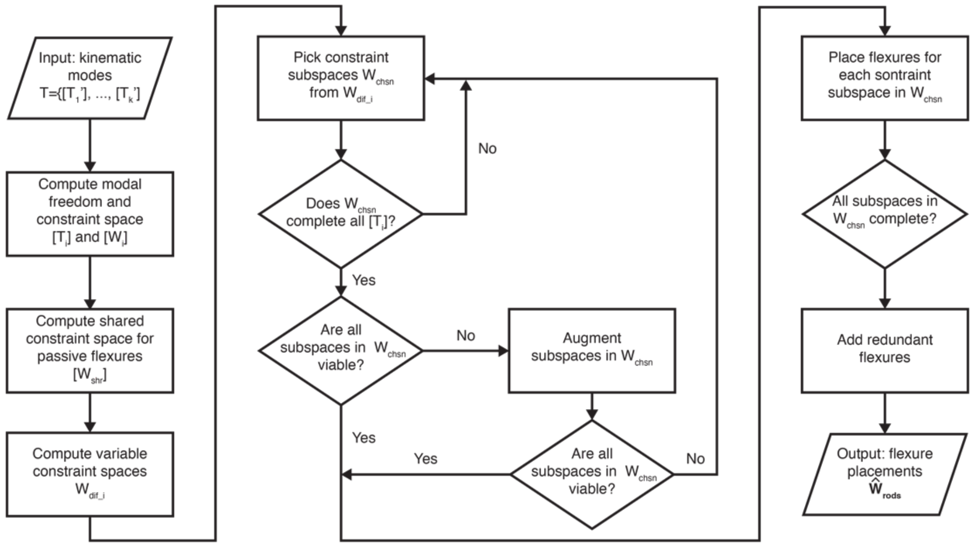

Figure S8. Algorithm for designing kinematically reconfigurable compliant metastructure joints.

The design process is divided into the following steps:

Step 1. Compute kinematic modes' freedom and constraint spaces.
Calculate each kinematic mode's corresponding freedom and constraint space using eq. 5 and eq. 7. Given a kinematic mode $[T_i'] \in T$, the freedom space $[T_i]$ can be found by computing:

$$[T_i] = \mathcal{N}(\mathcal{N}([T_i'])) \quad \text{(eq. 16)}$$

and the corresponding constraint space can be calculated using

$$[W_i] = \mathcal{N}\left([T_i]^T \begin{bmatrix} 0 & I \\ I & 0 \end{bmatrix}\right) \quad \text{(eq. 17)}$$

The derived spaces $[T_i]$ and $[W_i]$ will be used in later steps.

Step 2. Compute conventional flexure placements.
The all-intersection represents a constraint subspace $[W_{shr}]$ shared by all kinematic modes. Rods lying on this subspace are needed to enable all kinematic modes and, thus, are not required to be reconfigurable. The all-intersection of a device containing k kinematic modes is computed by

$$[W_{shr}] = \cap_{i=1}^{k}[W_i] \quad \text{(eq. 18)}$$

Noticeably, certain combinations of kinematic modes may lead to a shared constraint space that is unviable (e.g., the 6-DOF device). In this case, no conventional flexures should be used, or the kinematic modes will be over-constrained, and $[W_{shr}] = [0]$ is superimposed. In addition to the flexures added during the design process, any existing flexures and articulated joints (e.g., the wearer's wrist in Fig. 2a) also count toward this constraint space. Thus, it is possible to complete the shared constraint space without adding additional flexures, which is common in the design of wearable devices where the human skeleton readily and exactly constrains the kinematics.

Step 3. Finding variable constraint subspaces

To enable and exactly constrain kinematic mode i, stiffness-changing flexural rods should be placed in the subspace difference $[W_{dif\_i}]$ between $[W_{shr}]$ and $[W_i]$, such that the stiffened rods together with the conventional flexures complete $[W_i]$. I.e.,

$$[W_i] = [W_{shr}] \cup [W_{dif\_i}] \therefore [W_{dif\_i}] = [W_i] \backslash [W_{shr}] \quad \text{(eq. 19)}$$

Additionally, several difference subspaces may also share a constraint space larger than $[W_{shr}]$; stiffness-changing flexures placed in such spaces can be shared among several kinematic modes, and the reconfigurable device can be completed without adding stiffness-changing flexure to each and every difference subspace. The constraint subspace $[W_{\mathbf{A}}]$ shared by kinematic modes $A \subset T$ and no others (i.e., exclusively shared by modes in A) can be found by the following equation:

$$[W_{\mathbf{A}}] = \cap_{i \in \mathbf{A}}^{k} [W_i] \setminus \cup_{j \notin \mathbf{A}}^{k} [W_j] \quad \text{(eq. 20)}$$

For a device with k kinematic modes, there are potentially $2^k - 2$ constraint subspaces shared by different subsets of kinematic modes (minus the all-intersect and the constraint subspace that over-constrains all modes). However, some subspaces may produce an empty intersection and thus can be omitted. We call the set of all intersected subspaces W_{sub} in the following steps.

Step 4. Selecting variable constraint subspaces.

A minimum of k shared constraint subspaces should be chosen for placing stiffness-changing flexural rods. The chosen subspaces W_{chsn} should meet two conditions: for a kinematic mode i,

those included by $[W_i]$ should complete $[W_i]$:

$$[W_i] \leftrightarrow \cup_j [W_j] \, \forall [W_j] \in W_{chsn} \rightarrow [W_j] \subseteq [W_i] \quad \text{(eq. 21)}$$

Those not included by $[W_i]$ should complete $\mathcal{N}([W_i])^T$:

$$\mathcal{N}([W_i])^T \leftrightarrow \cup_j [W_j] \, \forall [W_j] \in W_{chsn} \rightarrow [W_j] \nsubseteq [W_i] \quad \text{(eq. 22)}$$

The former condition ensures a kinematic mode is exactly constrained when it's enabled, and the latter condition makes sure its kinematic freedom $[W_i]$ can be completely disabled in the other

kinematic modes. Additionally, W_{chsn} should always include $[W_{shr}]$ if it is viable.

It is worth mentioning that any combination of W_{chsn} is valid as long as eq. 21 and eq. 22 are met, which provides flexibility when designing the device (e.g., avoiding rod cluttering and collision, achieving targeted stiffness, and aesthetics). More information is provided in Section 2.6.

Step 5. Placing nonredundant flexures

Flexural rods should be added according to and complete each subspace in W_{chsn}. Note that some selected constraint subspaces may be unviable. In that case, such constraint subspace $[W_j]$ shared by kinematic modes $A_j \subset T$ can be augmented (unioned) with another constraint subspace $[W_{aug}]$ in W_{chsn} if the kinematic modes A_{aug} that intersected into $[W_{aug}]$ is a strict superset of A_j (i.e., $A_{aug} \subset A_j$). See section 2.6 for more details.

Step 6. Adding redundant flexures

In addition to the nonredundant flexures required to exactly constrain and enable each targeted kinematic mode, additional flexures may also be added to the device to achieve targeted performances. For the considerations when adding variable flexural rods, see Section 2.7 and Section 3.1 for more detail.

Figure S9 provides an exemplary design process following the algorithm, using the wrist joint design as an example. Each input kinematic mode is represented as screw linear spaces in steps 1 to 4. Then, the design was modeled in steps 5 and 6 by placing flexural rods and replacing passive rods with the human skeletal structure.

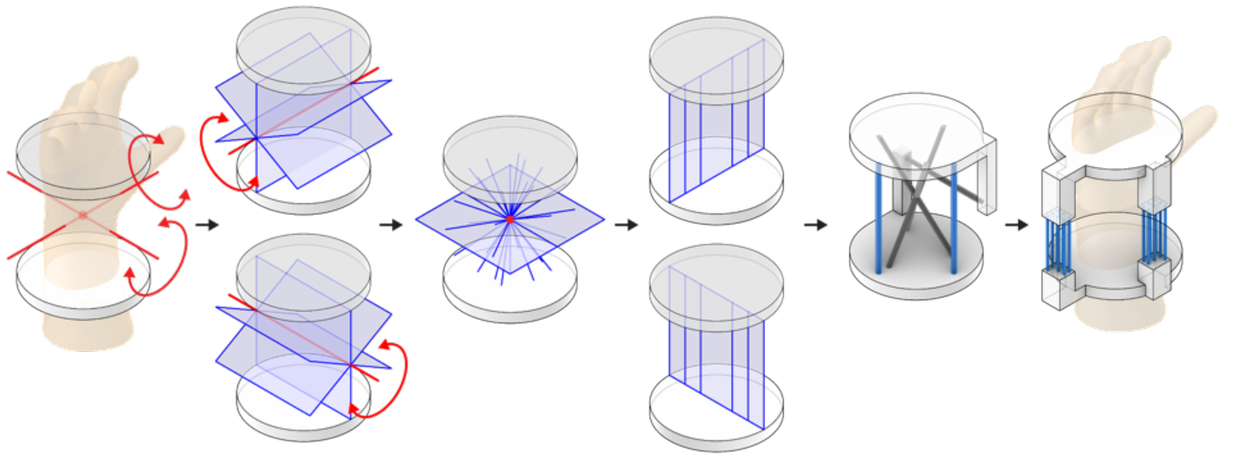

Figure S9. A step-by-step design example of the kinematics design algorithm using the wrist joint as an example.

2.4 Determining Flexure State for Kinematic Modes

A stiffness-changing flexural rod's state under a kinematic mode i can be determined by comparing its wrench vector $\widehat{\mathbf{W}}_{\mathbf{rod}}$ against the kinematic mode's constraint space $[\mathrm{W_i}]$. $\widehat{\mathbf{W}}_{\mathbf{rod}}$ should be softened if it is not spanned by $[\mathrm{W_i}]$. This check can be performed using the following formula:

$$\mathcal{N}([\mathrm{W_i}])\widehat{\mathbf{W}}_{\mathbf{rod}} = \widehat{\mathbf{0}} \qquad \text{(eq. 23)}$$

If the formula evaluates to false, then the wrench vector is not included in $[\mathrm{W_i}]$ and is an over-constraining element; thus, should be softened to enable the targeted kinematic mode. Conversely, if the formula evaluates to true, the rod can be left stiffened without compromising the targeted kinematics. Such flexures can also be softened to provide a lower stiffness along the enabled DOF, but it should be noted that the constraint space formed by stiff flexure $[\mathrm{W_{stf}}]$ should complete $[\mathrm{W_i}]$ to exactly constrain the mode. Thus, the following conditions are required:

$$\mathcal{N}([\mathrm{W_i}])[\mathrm{W_{stf}}] = [0] \qquad \text{(eq. 24)}$$

$$\mathcal{N}([\mathrm{W_{stf}}])[\mathrm{W_i}] = [0] \qquad \text{(eq. 25)}$$

2.5 Representing Constraint Subspaces Using Venn Diagrams

The constraint subspaces in steps 2 and 3 of the algorithm can be represented as a Venn diagram to show their relation with kinematic modes. Such a diagram is helpful during the rational design process to help users identify and strategize flexure placements and modal reconfigurations. The constituent shapes (e.g., circle) represent the kinematic modes' constraint spaces, and subspaces intersected by shapes are shared among them. In particular, the center of a Venn diagram is the all-intersection and represents $[W_{shr}]$, whereas other segmented regions represent constraint spaces shared by the overlapping kinematic modes (i.e., W_{sub}).

The spaces encircled by a kinematic mode i are spanned by its constraint space and can be stiffened under mode i. Conversely, the subspaces not included by a kinematic mode will over-constrain it and should be softened to enable the mode. In step 4 of the algorithm, the two conditions for choosing W_{chsn} can also be interpreted on the Venn diagram: the union of chosen subspaces encircled by kinematic mode i should complete $[W_i]$, and the ones outside should complete $\mathcal{N}([W_i])^T$.

2.6 Principles of Selecting Variable Constraint Subspaces

The conditions described in step 4 of the algorithm are the minimum requirements for designing a multimodal kinematic device. We note that some design properties or objectives (e.g., reducing the number of rods) can be obtained through tactful choosing of W_{chsn} and to avoid certain mechanical design issues.

Designing a device with fewer rods can be achieved by prioritizing W_{chsn} on most-shared subspaces, such that rods placed in those can be shared by as many kinematic modes as possible and require fewer stiffness-changing flexures to complete the design. For a device with k kinematic modes, prioritizing picking subspaces shared by a descending number of modes (i.e., $k-1$, $k-2$, …, 1.) will be an efficient way of choosing constraint subspaces. Each descending level is shared by fewer kinematic modes and should only be chosen when the preceding ones do not complete the modes' constraint subspaces.

Constraint space intersections may also yield unviable subspaces (e.g., without a directional component) or cause mechanical design issues (e.g., rods cluttering and overlapping). In this case, the subspaces can be augmented to provide more design freedom. An unviable constraint subspace $[W_{\mathbf{A}}]$ intersected by a subset of modes A can be expanded into $[W'_{\mathbf{A}}]$ by lifting the difference operator in eq. 20:

$$[W'_{\mathbf{A}}] = \cap^{k}_{j\in\mathbf{A}}[W_j] \quad \text{(eq. 26)}$$

such that $[W'_{\mathbf{A}}]$ navigates a larger linear subspace. This augmentation, in turn, provides more freedom for placing rods. Additionally, we may also re-write $[W'_{\mathbf{A}}]$ as the intersection of $[W'_{\mathbf{A\_1}}]$

and $[W'_{\mathbf{A\_2}}]$ with their corresponding intersection mode subsets, A_1 and A_2, respectively, meeting the following condition:

$$[W'_A] = [W'_{A\_1}] \cap [W'_{A\_2}], \text{where } A_1 \cup A_2 = A, A_1 \subset A \;\&\; A_2 \subset A \qquad \text{(eq. 27)}$$

This can be interpreted as dividing A into two subsets with lower cardinality (i.e., the number of modes sharing a constraint subspace), each containing fewer kinematic modes. After applying eq. 27, the products $[W'_{\mathbf{A\_1}}]$ and $[W'_{\mathbf{A\_2}}]$ navigate a larger constraint subspace than $[W'_{\mathbf{A}}]$ since it is the intersection of fewer kinematic modes. This way, both $[W'_{\mathbf{A\_1}}]$ and $[W'_{\mathbf{A\_2}}]$ should be softened to enable a kinematic mode $i \notin A$, and if mode $i \in A$, $[W'_{\mathbf{A\_j}}]$ should be softened if $i \notin A_j$. More, $[W'_{\mathbf{A}}]$ can be re-written into the intersection of any number of constraint subspaces, and the condition described in 26 is expanded into

$$[W'_A] = \cap_i [W'_{A\_i}], \text{where } \cup_i A_i = A \;\&\; A_i \subset A \forall i \qquad \text{(eq. 28)}$$

Finally, constraint subspace augmentation can also be addressed on a vector level. If a constraint subspace $[W_{\mathbf{A}}]$ is missing or lacking directional components, it is advised to augment it using eq. 26 and eq. 27 to produce constraint subspaces that afford valid rod placements. Alternatively, if $[W_{\mathbf{A}}]$ leads to cluttered or mechanically unviable rod placements, it should be augmented with a positional component.

2.7 Principles of Placing Flexural Rods

When placing flexural rods, in addition to common mechanical design considerations (e.g., avoiding collision, delivering targeted stiffness), the rod's direction should also be taken into notice. Flexural rods undergo different types of loads depending on whether they are in (i.e., as conventional flexures) or outside of (i.e., as kinematically locking elements or stiffness-changing flexures) a constraint space with respect to a motion. Rods placed in a kinematic mode's constraint space will dominantly bend to enable that motion. By contrast, rods placed outside the constraint space are chiefly subjected to axial loads. Therefore, the orientation of these rods should be considered when they exhibit drastically different extensions and compression deformability, such as the epoxy rods used in this work. In particular, the stiffness-changing rods can only compress, not extend. Thus, an additional check is required to ensure all rods are subjected to compressive loads under a prescribed motion. This check can be performed by using

$$D = \left(\hat{\mathbf{n}}_{\mathbf{axis}} \times (\hat{\mathbf{r}}_{\mathbf{rod}} - \hat{\mathbf{c}}_{\mathbf{axis}})\right) \cdot \hat{\mathbf{n}}_{\mathbf{rod}} \qquad \text{(eq. 29)}$$

for rotational and

$$D = \hat{\mathbf{n}}_{\mathbf{axis}} \cdot \hat{\mathbf{n}}_{\mathbf{rod}} \qquad \text{(eq. 30)}$$

for translational motions, where D is a scalar indicator of a rod's direction with respect to the

motion, $\hat{\mathbf{n}}_{\mathbf{axis}}$ and $\hat{\mathbf{n}}_{\mathbf{rod}}$ are vectors along and $\hat{\mathbf{c}}_{\mathbf{axis}}$ and $\hat{\mathbf{r}}_{\mathbf{rod}}$ are reference points on the motional and rod axis, respectively. In particular, $\hat{\mathbf{n}}_{\mathbf{rod}}$ should always point from the fixed base stage to the free-moving stage. The rod is subjected to compression if $D < 0$ and extension if $D > 0$. $D = 0$ indicates that the rod lies in the motion's constraint space. Note that this check is only required by flexures that are not extensible. If the flexures are instead allowed to deform in both directions, the check can be omitted.

Supplementary Table S1: List of notations used throughout this work.

Scalars	
p	Twist vector pitch
q	Wrench vector pitch
ω	Twist vector angular velocity
v	Pure translational twist vector velocity
f	Wrench vector force magnitude
n	A compliant mechanism's degree of freedom
m	A compliant mechanism's degree of constraint
D	Rod and motion direction indicator
K	Stiffness value
r	Flexural rod radius
l	Flexural rod length
θ, φ	Angular parameters
Vectors	
Symbol	Use
$\hat{\mathbf{T}}$	6×1 Twist screw vector
$\hat{\mathbf{W}}$	6×1 Wrench screw vector

$\hat{\mathbf{n}}$	3×1 Axis direction unit vector
$\hat{\mathbf{c}}$	3×1 Twist screw vector reference point on axis coordinates
$\hat{\mathbf{r}}$	3×1 Wrench screw vector reference point on axis coordinates
$\hat{\mathbf{0}}$	Zero vector
$\hat{\mathbf{J}}_{\mathbf{f}}$	6×1 Joint instantaneous freedom velocity
$\hat{\mathbf{J}}_{\mathbf{c}}$	6×1 Joint instantaneous constraint force
$\hat{\mathbf{x}}$	Joint freedom space velocity parameter vector
$\hat{\mathbf{y}}$	Joint constraint space force parameter vector
$\|\hat{\mathbf{a}}\|_2$	L2 norm of $\hat{\mathbf{a}}$
Linear systems	
$[\mathrm{T}]$	Freedom subspace
$[\mathrm{W}]$	Constraint subspace
$[\mathrm{K}]$	Stiffness matrix
$[0]$	Zero matrix
$[\mathrm{Ad}]$	Adjoint transformation matrix
$\mathcal{N}([\mathrm{A}])$	The nullity (kernel) of linear space $[\mathrm{A}]$
$\mathrm{Rank}([\mathrm{A}])$	The rank of a linear space $[\mathrm{A}]$
Boolean operations	
$\cup$	Union of two sets or linear spaces
$\cap$	Intersection of sets or linear spaces
$\setminus$	Difference between sets or linear spaces
$\bigcup_a$	Consecutive Union (of sets or linear spaces) over a
$\bigcap_a$	Consecutive Intersection (of sets or linear spaces) over a

Sets	
A	A set
⊂	Strict subset of
⊆	Subset of
Logic	
↔	Linear system equivalence.
⇔	Conditional equivalence
→	If condition (i.e., left-hand side if the right-hand side is true)

Supplementary Note 3: Stiffness Model and Finite Element Simulation

3.1 Analytical Stiffness Model

The device's stiffness is also a pivotal part of the design process, especially when designing for locking motions under an expected load. For this reason, we provide a summary of design parameters that affect a compliant mechanism's stiffness with respect to a motion. More detailed analysis can be found in the literature[16].

A flexure's deformation twist $\widehat{\mathbf{T}}$ and reaction wrench $\widehat{\mathbf{W}}$ are related by

$$\widehat{\mathbf{W}} = [\mathrm{K}]\widehat{\mathbf{T}} \qquad \text{(eq. 31)}$$

For a compliant joint comprising parallel flexures, the joint's stiffness matrix $[\mathrm{K}_{\mathrm{joint}}]$ can be modeled as the sum of its parallel flexures:

$$[\mathrm{K}_{\mathrm{joint}}] = \Sigma_{\mathrm{i}}[\mathrm{K}_{\mathrm{i}}] \qquad \text{(eq. 32)}$$

Where $[\mathrm{K}_{\mathrm{i}}]$ is the stiffness matrix of flexure i within the parallel flexure joint. Note that all $[\mathrm{K}_{\mathrm{i}}]$ must share the same reference frame in space.

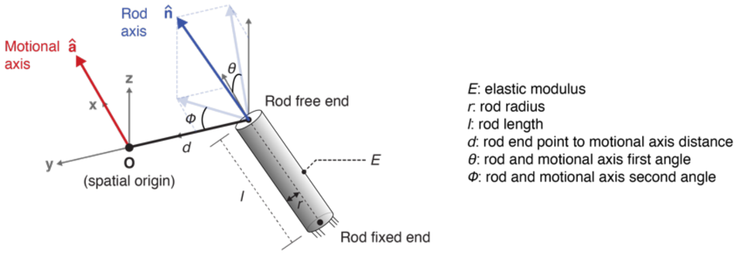

Figure S10. The flexural rod and motional axis parameterization used by the analytical stiffness model.

Assuming a cylindrical flexural rod of radius r and length l (Fig. S10). Its relative position with respect to a motional axis $\hat{\mathbf{a}}$ can be described with three parameters d, θ, and φ, where d is the minimal distance between the rod's axis $\hat{\mathbf{n}}$ and the motional axis (Fig. 2b-ix). The angular parameters θ and φ describe the rod vector's direction with respect to the motion axis. The material constituting the rod is assumed to have an elastic modulus E and shear modulus G. The flexure's second moment of inertia $I = \pi r^4/4$ and torsion constant $I = \pi r^4/2$ are then functions of its geometric parameters.

Based on the Euler beam theory, the flexure's stiffness against a motion is proportional to r and inversely proportional to l. On the other hand, the relative position also affects a flexure's stiffness

contribution to a motion of interest. Following the model established by Su et al.[16], an x-axis-aligned flexural rod's stiffness matrix $[\mathrm{K_c}]$ at the center middle of the flexure can be expressed as:

$$[\mathrm{K_c}] = \begin{bmatrix} 0 & \mathrm{I} \\ \mathrm{I} & 0 \end{bmatrix} \begin{bmatrix} K_T & 0 & 0 & 0 & 0 & 0 \\ 0 & K_B & 0 & 0 & 0 & 0 \\ 0 & 0 & K_B & 0 & 0 & 0 \\ 0 & 0 & 0 & K_A & 0 & 0 \\ 0 & 0 & 0 & 0 & K_L & 0 \\ 0 & 0 & 0 & 0 & 0 & K_L \end{bmatrix} \qquad \text{(eq. 33)}$$

Where $K_A = EA/l$ and $K_L = 12EI/l^3$ are the rod's stiffness against axial and lateral translations, respectively, and $K_T = GJ/l$ and $K_B = EI/l$ are the rod's torsional and bending stiffness against rotations about and perpendicular to its axis. The swap on the left operator was added because Su et al.[16] used a different definition for wrench vectors (i.e., force vector preceding moment vector). Given $[\mathrm{K_c}]$, the stiffness matrix $[\mathrm{K}]$ at the free end of the flexure can be found by applying adjoint transformation:

$$[\mathrm{K}] = \begin{bmatrix} 0 & \mathrm{I} \\ \mathrm{I} & 0 \end{bmatrix} [\mathrm{Ad_c}][\mathrm{K_c}][\mathrm{Ad_c}]^{-1} \qquad \text{(eq. 34)}$$

Where $[\mathrm{Ad_c}]$ is the adjoint transformation matrix[16] from the rod's middle to the free end frame, which can be expressed as:

$$[\mathrm{Ad_c}] = \begin{bmatrix} \mathrm{I} & 0 \\ \mathrm{D_C} & \mathrm{I} \end{bmatrix}, \mathrm{D_C} = \begin{bmatrix} 0 & 0 & 0 \\ 0 & 0 & \frac{1}{2} \\ 0 & -\frac{1}{2} & 0 \end{bmatrix} \qquad \text{(eq. 35)}$$

After substitution, we then get:

$$[\mathrm{K}] = \begin{bmatrix} 0 & 0 & 0 & K_A & 0 & 0 \\ 0 & 0 & -\frac{K_L l}{2} & 0 & K_L & 0 \\ 0 & \frac{K_L l}{2} & 0 & 0 & 0 & K_L \\ K_T & 0 & 0 & 0 & 0 & 0 \\ 0 & K_B + \frac{K_L l^2}{4} & 0 & 0 & 0 & \frac{K_L l}{2} \\ 0 & 0 & K_B + \frac{K_L l^2}{4} & 0 & -\frac{K_L l}{2} & 0 \end{bmatrix} \qquad \text{(eq. 36)}$$

Moreover, assuming the motion is x-axis-aligned and passes through the spatial origin. The flexure's endpoint is located at $(0, -d, 0)$, and the flexure's stiffness matrix $[\mathrm{K}']$ at the motion axis frame can be found by

$$[\mathrm{K}'] = \begin{bmatrix} 0 & \mathrm{I} \\ \mathrm{I} & 0 \end{bmatrix} [\mathrm{Ad}'][\mathrm{K}][\mathrm{Ad}']^{-1} \qquad \text{(eq. 37)}$$

Where [Ad′] is the adjoint transformation matrix between the flexure's free end and the motional axis frame, defined as:

$$[\mathrm{Ad}'] = \begin{bmatrix} \mathrm{R} & 0 \\ \mathrm{DR} & \mathrm{R} \end{bmatrix}, \mathrm{R} = \begin{bmatrix} \cos\theta\cos\varphi & -\cos\theta\sin\varphi & \sin\theta \\ \sin\varphi & \cos\theta & 0 \\ -\sin\theta\cos\varphi & \sin\theta\sin\varphi & \cos\theta \end{bmatrix}, \mathrm{D} = \begin{bmatrix} 0 & 0 & -d \\ 0 & 0 & 0 \\ d & 0 & 0 \end{bmatrix} \quad \text{(eq. 38)}$$

Consequently, in the motional axis frame, the reaction wrench $\widehat{\mathbf{W}}'$ resulted from a displacement twist $\widehat{\mathbf{T}}'$ is then found by plugging [K′] and $\widehat{\mathbf{T}}'$ into eq. 31.

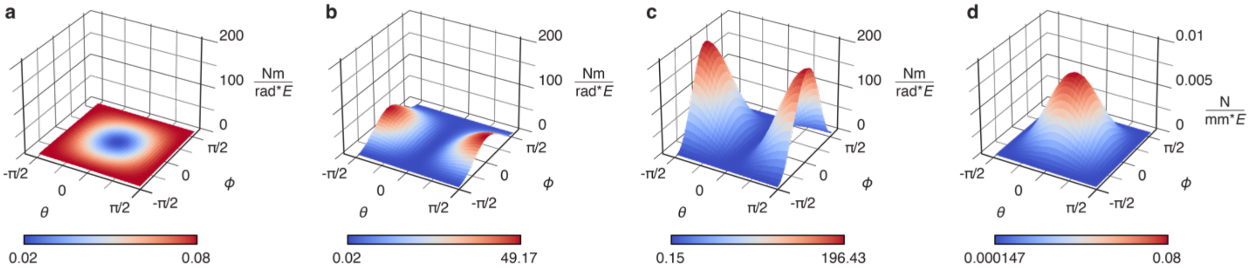

Figure S11. Stiffness and design parameter sweep using the analytical model, normalized by the material's elastic modulus. *This study assumes the flexural rod to have* $r = 1$ *mm,* $l = 40$ *mm, and a Poisson's ratio of* 0.3. *Subfigures **a-c** shows the plots for a rotational motion at* $d = 0$ ***(a)***, 25 ***(b)***, *and* 50 ***(c)*** *mm, whereas subfigure **d** shows the stiffness plot against a translational motion.*

Note that both $\widehat{W}'$ and $\widehat{T}'$ are also defined in the motional axis frame. Therefore, one could substitute $\widehat{T}'$ with a unit rotation or translation vector to calculate reaction forces. The moment M_x reacting about a unit rotation is then computed as

$$M_x = (\cos^2\theta\cos^2\varphi)K_T + (1-\cos^2\theta\cos^2\varphi)K_B + d^2\big((\sin^2\theta\cos^2\varphi)K_A + (1-\sin^2\theta\cos^2\varphi)K_L\big) + \left(\frac{l^2(1-\cos^2\theta\cos^2\varphi)}{4} + dl\sin\varphi\right)K_L \quad \text{(eq. 39)}$$

From this definition, we can find that the rod primarily bends and twists in reaction to the rotation when its axis intercepts the motional axis (i.e., $d = 0$ or $\theta = 0$; see also Fig. S11a-c). In this case, the rod also falls into the rotation's corresponding constraint space. Noticeably, when the rod is placed outside of the constraint space (i.e., $d \neq 0$) and not parallel with $\hat{\mathbf{a}}$ (i.e., $\theta \neq 0$), the rod is subjected to additional translational displacements. In particular, the load increases quadratically proportional to d. Due to the high axial stiffness (i.e., $K_A \gg K_L, K_B, K_T$), the rod becomes acutely more resistant to rotation. On the other hand, θ and φ together determine the tradeoff between the rod's axial and lateral translations and rotations. The rod is primarily subjected to axial rotation (torsion) and lateral translation when it is more aligned with $\hat{\mathbf{a}}$, and lateral bending and axial translation become more dominant as the rod's axis $\hat{\mathbf{n}}$ deviates from the rotation axis $\hat{\mathbf{a}}$. Given the rods' slender aspect ratio, the flexure becomes acutely stiffer against the rotation.

Similarly, the force F_x reacting against and a unit translation is computed as

$$F_x = (\cos^2\theta\cos^2\varphi)K_A + (1 - \cos^2\theta\cos^2\varphi)K_L \quad \text{(eq. 40)}$$

Where d no longer determines the rod's deformation mode. This observation aligns with the intuition that the free stage's rigid body translation applies the same displacement regardless of the flexure's position. Still, θ and φ determine the rod's deformation modes. When θ and φ are small (i.e., rod aligned with translation axis), the flexure primarily undergoes axial displacement and is stiff (Fig. 11d). However, when the rod is placed perpendicular to the translational axis (i.e., $\theta = \pi/2$ or $\theta = 3\pi/2$), the stiffness is minimized as K_A is canceled out.

The above equations and intuitions can be leveraged in the design process to adjust the kinematic devices' performance. In particular, taking partial derivatives of the above equations can help to find the parameters required to increase or decrease the device's stiffness. For instance, if the locking effects provided by a stiffness-changing flexure are lower than the design criteria, one may consider increasing its distance to the motional axis to increase the locking effect acutely. Similarly, to increase the locking stiffness against a translational motion, we may orient the rod more perpendicular to the direction of translation, reducing the contribution of the K_A term. In addition to adjusting the flexures' geometric parameters and relative positions, the stiffness of a parallel flexure joint can also be tuned by adding and removing rods. These design decisions are demonstrated in the Device Design section.

It is worth mentioning that a (stiffness-changing) flexure's buckling criterion is also affected by its placement with respect to a motion. When designing a mode to lock a motion, the buckling criterion must be higher than the expected loads. Yet, finding the buckling criterion of a flexure undergoing complex loads requires elliptic integrals[17], and its integration for screw algebra-based compliant mechanism design leads to a highly nonlinear optimization process, making analytical solutions nearly impossible. Hence, we use FE simulations (see next section) to validate a design's buckling behavior against expected loads.

3.2 Finite Element Simulation

We further perform finite element (FE) simulations to predict the nonlinear stiffness of the devices under large deformation and rotation to verify and iterate the design's performance before fabricating them for mechanical tests. For simplicity, the devices are assumed to be made of isotropic elastic material models (Table S2). The stiffness-changing flexures are also modeled as homogeneous bodies without explicit heating wires and epoxy interactions. Yet, they are modeled with an effective modulus representing the combined stiffness of both components. To achieve this, we fabricated 50 mm long samples ($n = 3$) with outer diameter of 1.5 mm and 2 mm and subjected them to axial compression under stiffened and softened states to acquire their load-displacement curves (see Section 6.3). The elastic modulus is then calculated from the pre-buckling linear region by normalizing against the rods' geometric parameters. The stages are

modeled without through holes and simplified by removing mechanical features that provide minimal structural functions (e.g., wiring guides and service panels). Bonded contacts are applied to the interface between the flexures and the stages to model their connection.

We used Ansys Mechanical to conduct the FE simulations using the Static Structural implicit solver. We enabled large deflection and automatic time stepping in the solver controls to accommodate flexure buckling. The device geometries are imported as STEP files and meshed in the Ansys Mechanical interface. Due to their distinct deformation behaviors, we used different mesh settings for the stages and flexures. The stages are meshed with default settings and an element size of 1 mm using tetrahedral (Tet10) elements. Conversely, the flexures are meshed by dividing the circumference into sixteen control points and with a 0.5 mm face-sizing on the cylindrical surface, leading to a hybrid mesh consisting of hexahedron (Hex20) and wedge prism (Wed15) elements.

The loads and boundary conditions are applied according to the context of each device's design and mechanical test setup (Fig. S12, S13, S14). Table S3 provides a summary of the settings for each simulation model setup. In brief, all models are applied with a fixed boundary condition at the fixed stage and prescribed displacements at the free end. The simulated force or moment reactions are recorded and compared with experimental measurements. For the wearable device, the external kinematic constraints created by the human skeleton are modeled as remote rotational pivot points and used by the displacement loads. It is worth noting that during device design iterations, the rigid stages are not fully modeled for complete analysis. We apply a remote rigid body connection between the flexural rods' ends and the boundary condition surface to simulate flexural rod performances.

The FE simulations were used to iterate our designs to make sure they satisfy the targeted design criteria (e.g., stiffness, buckling loads). To iterate a design, we check the simulation results to identify the difference between the current and targeted performance and use the analytical stiffness model to identify the parameters affecting the performance and the gradient of changes. Once a design is modified, we subject the new design to FE simulations to evaluate its improved performance until the targeted criteria are satisfied. Additionally, the FE simulations were also used to iterate and make sure the rigid stages remained sufficiently rigid and stable against the expected loads.

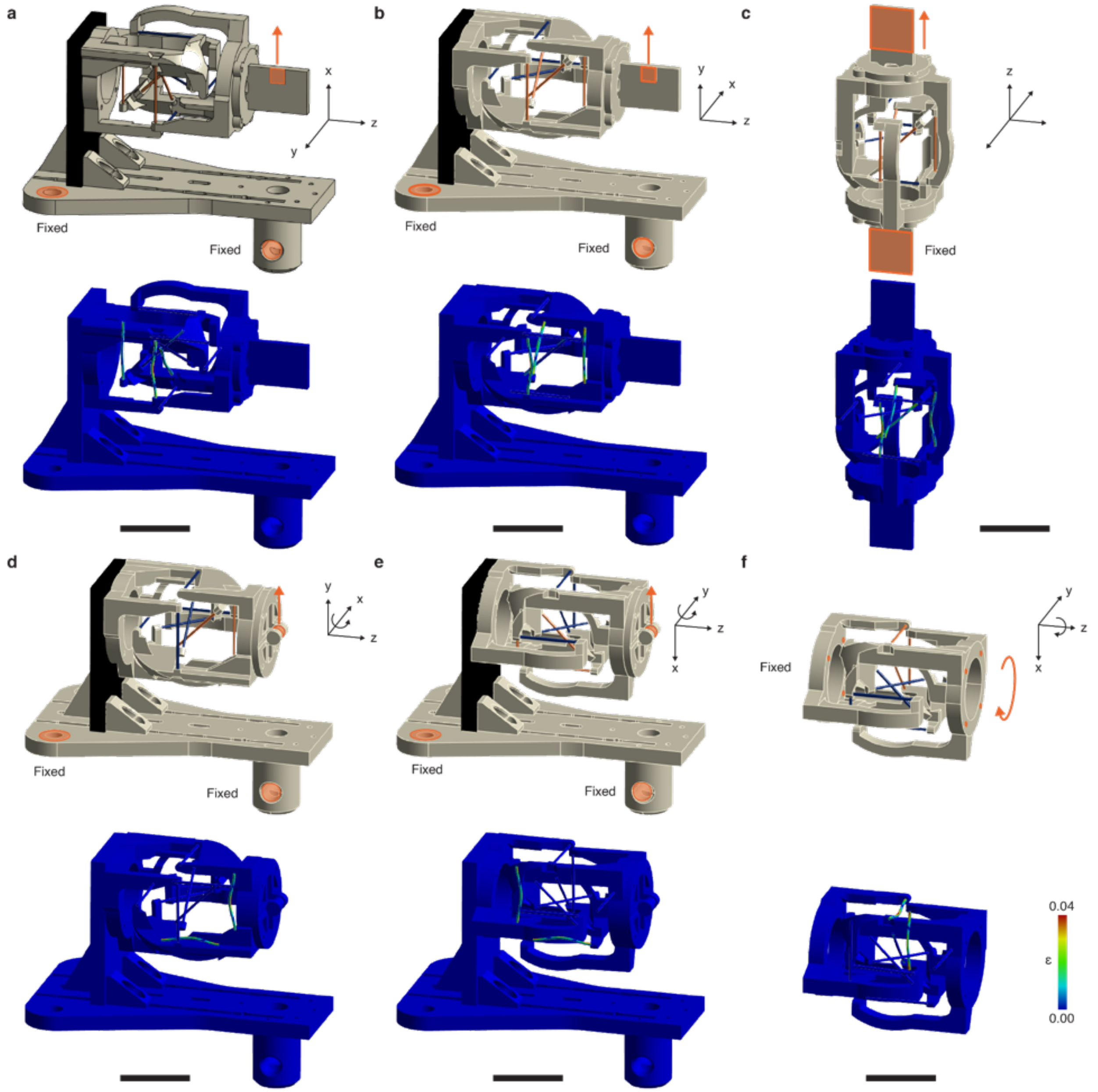

Figure S12. 6-DOF device FEA setup and simulation result. *The top rows in each subfigure show the FEA setup. The arrows show displacement load application sites. The bottom row shows the simulation results for each unlocked state:* ***a****, x-translation;* ***b****, y-translation;* ***c****, z-translation;* ***d****, x-rotation;* ***e****, y-rotation;* ***f****, z-rotation. Colors indicate material assignment: gray, PLA; black, aluminum; blue, stiffness-changing flexures (RT); orange, Stiffness-changing flexures (54℃). Scale bar, 50 mm.*

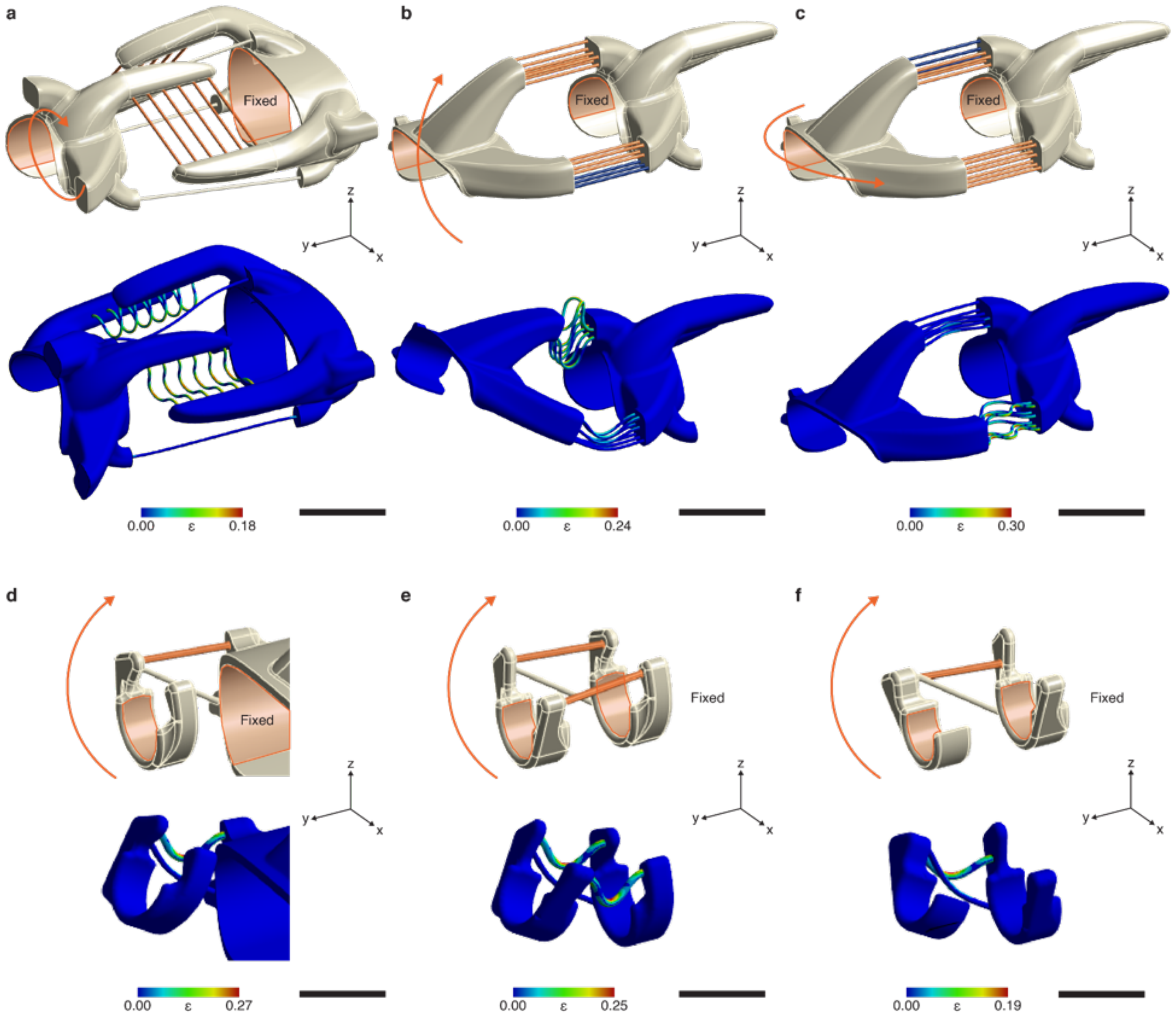

Figure S13. Arm-wearable device FEA setup and simulation result. *The top rows in each subfigure show the FEA setup. The arrows show displacement load application sites. The bottom row shows the simulation results for each of the joints in their unlocked states:* ***a****, forearm pronation;* ***b****, wrist flexion;* ***c****, wrist deviation;* ***d****, MP flexion;* ***e****, PIP flexion;* ***f****, DIP flexion. Colors indicate material assignment: gray, Formlabs white resin; black, aluminum; blue, stiffness-changing flexures (RT); orange, Stiffness-changing flexures (54℃). Subfigure a-c scale bar, 50 mm. Subfigure d-f scale bar, 25 mm.*

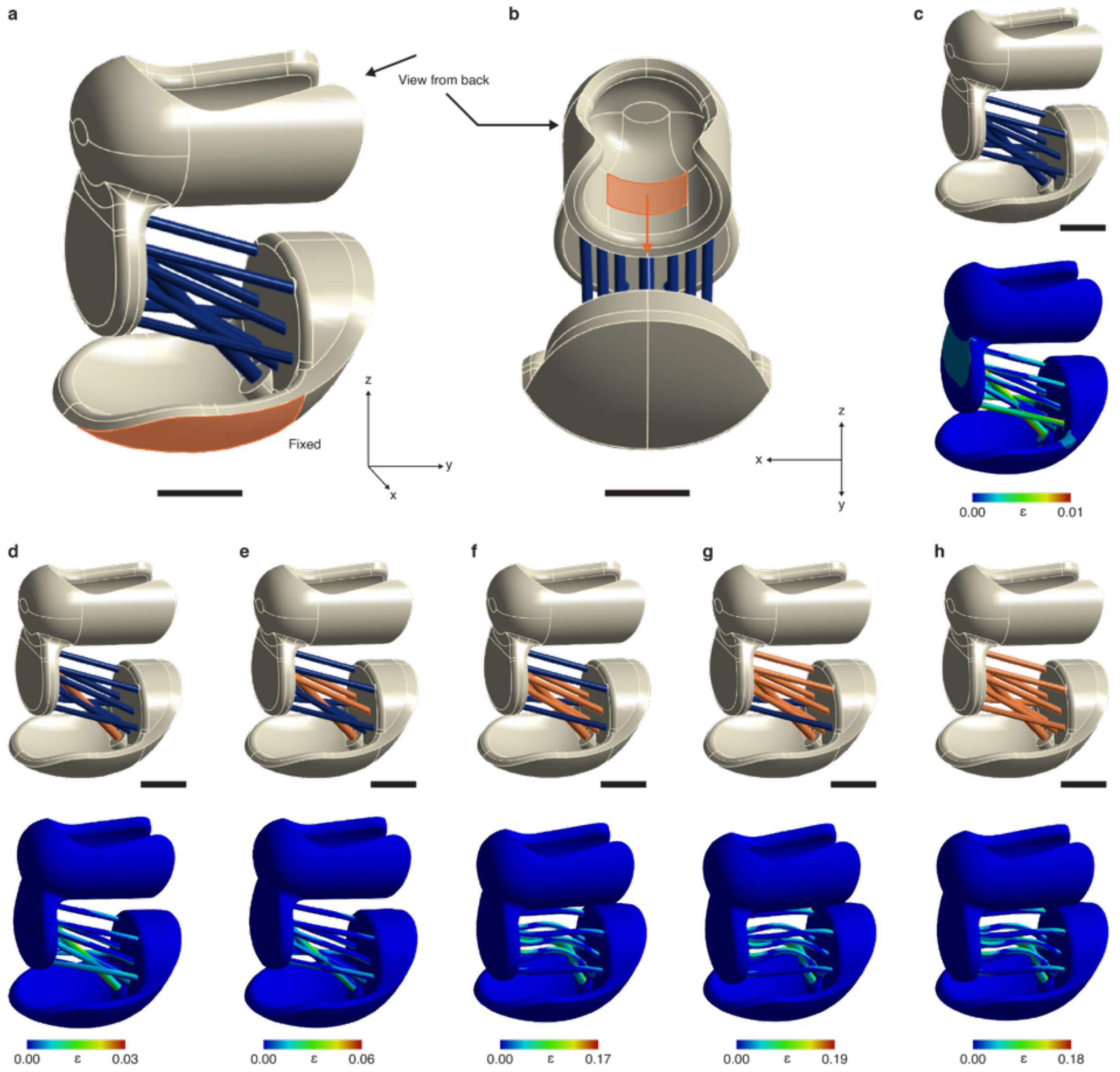

Figure S14. Haptic thimble FEA setup and simulation result. a*, the device's fixed-end application.* ***b****, the device's displacement load (negative z) application shown at a different view angle than in subfigure a. Subfigure c-h shows the FEA setup (top row) and simulation results (bottom row) in different device configurations:* ***c****, [0, 0, 0, 0, 0];* ***d****, [0, 0, 0, 0, 1];* ***e****, [0, 1, 0, 0, 1];* ***f****, [0, 1, 1, 0, 1];* ***g****, [1, 1, 1, 0, 1];* ***h****, [1, 1, 1, 1, 1]. Colors indicate material assignment: gray, Formlabs white resin; black, aluminum; blue, stiffness-changing flexures (RT); orange, Stiffness-changing flexures (54℃). Scale bar, 10 mm.*

Supplementary Table S2. FE simulation material definitions.

Material	Elastic modulus	Poisson's ratio	Applied to
Polyacrylic acid (PLA)	3204 MPa	0.3	Rigid stages, passive flexures
Formlabs white resin	2800 MPa	0.3	Rigid stages
Aluminum	68 GPa	0.33	6-DOF device jig
Stiffness-changing flexures (OD 2 mm)	2958.4 MPa (RT), 152.02 MPa (54°C)	0.3	Stiffness-changing flexures
Stiffness-changing flexures (OD 1.5 mm)	2668.9 MPa (RT), 480.86 MPa (54°C)	0.3	Stiffness-changing flexures

Supplementary Table S3. FE simulation boundary conditions.

Device	Load type	Fixed condition	Displacement load	Load
6-DOF device	X, Y-translation	Interface between the jig and the Instron machine clamp: all six DOF	Interface between the jig and the load cell clamp:<br>Fixed DOF: all except for load	Displacement: 1 mm (Unlocked: 0.2 mm/step, locked: 0.02 mm/step)
	Z-translation	Interface between the jig and the Instron machine: all six DOF	Interface between the jig and the load cell clamp:<br>Fixed DOF: X_T, Y_T, X_R, Y_R, Z_R	Displacement: 1 mm (Unlocked: 0.2 mm/step, locked: 0.02 mm/step)
	X, Y-rotation	Interface between the jig and the Instron machine: all six DOF	Interface between jig the rotational bearing at the device free end:<br>Fixed DOF:<br>X_T, Y_R, Z_R (X rotation);<br>Y_T, X_R, Z_R (Y rotation)<br>Free DOF:<br>X_R, Z_T (X rotation);<br>Y_R, Z_T (Y rotation)	Displacement: 2 mm (Unlocked: 0.2 mm/step, locked: 0.02 mm/step)
	Z-rotation	Interface between the device and the jig: all six DOF	Interface between the device and the loading jig:<br>Fixed DOF: X_T, Y_T, Z_T, X_R, Y_R	Displacement: 2 mm (Unlocked: 0.2 mm/step, locked: 0.02 mm/step)
Wearable device (forearm)	Axial rotation (with remote point to specify axis of rotation)	Interface between the fixed stage and the wearer	Interface between the free stage and the wearer:<br>Fixed DOF: X_T, Y_T, Z_T, X_R, Z_R<br>Load DOF: Y_R	Displacement: 0.6 deg @ 0.02 deg/step (locked), 30 deg @ 0.2deg/step (unlocked)
Wearable device (wrist)	Flxion (with remote point to specify axis of rotation)	Interface between fixed stage and the wearer	Interface between the free stage and the wearer:<br>Fixed DOF: X_T, Y_T, Z_T, X_R, Z_R<br>Load DOF: Y_R	Displacement: 0.6 deg @ 0.02 deg/step (locked), 30 deg @ 0.2 deg/step (unlocked)
	Deviation (with remote point to specify axis of rotation)	Interface between fixed stage and the wearer	Interface between the free stage and the wearer:<br>Fixed DOF: X_T, Y_T, Z_T, X_R, Y_R<br>Load DOF: Z_R	Displacement: 0.6 deg @ 0.02 deg/step (locked), 30 deg @ 0.2 deg/step (unlocked)

Wearable device (finger joints)	Rotation (with remote point to specify axis of rotation)	Interface between fixed stage and the wearer	Interface between the free stage and the wearer:<br>Fixed DOF: X_T, Y_T, Z_T, Y_R, Z_R<br>Load DOF: X_R	Displacement: 0.6 deg @ 0.02 deg/step (locked), 30 deg @ 0.2 deg/step (unlocked)
Haptic thimble	All modes (with remote point to specify axis of rotation)	Interface between the device and the Instron machine: all six DOF	Interface between jig and load cell clamp:<br>Fixed DOF: X_T, Y_R, Z_R<br>Freed DOF: Y_T, X_R<br>Load DOF: Z_T	Displacement (all load applied at 0.02 mm/step): 0.6 mm([0, 0, 0, 0, 0]), 2 mm ([0, 0, 0, 0, 1]), 5 mm ([0, 1, 0, 0, 1], [0, 1,1, 0, 1], [1, 1, 1, 0, 1], [1, 1, 1, 1, 1])

Supplementary Note 4: 6-DOF Device Design

4.1 6-DOF Device: Design Summary

The 6-DOF device was intended to demonstrate the effectiveness of the multimodal kinematics algorithm in identifying flexure placements (Video S1). Each mode is defined by its kinematic specifications without a stiffness requirement. Therefore, this design example did not use the FE simulation and the analytical stiffness model in its iterations. However, we demonstrate that the algorithm and design rules can help designers create designs with certain qualities, such as avoiding flexure cluttering, symmetry, and more.

4.1 6-DOF Device: Rational Design of Flexure Placement

Step 1 & design specifications

The 6-DOF device was designed with a target length of 100 mm. Two rigid stage placeholders were added to signify screw hole positions for connection with test jigs. Six kinematic modes were specified as the input to the algorithm, each enabling a rigid body DOF in the 3D space (Fig. S15a). The device's center point was set as the rotation pivot and the spatial origin for calculations.

Steps 2 & 3

Figure S15b shows the constraint spaces resulting from the rational design algorithm. Detailed constraint subspaces are also documented in Table S4 for readers' reference. The kinematic mode specification led to an empty shared constraint subspace per Maxwell's equation of rigid body constraints. I.e., no constraints can be added if all six DOF should be enabled simultaneously. On the other hand, the constraint subspaces shared by the three translational DOF are also invalid since they led to subspaces with zero directional components where no flexural rods can be placed accordingly.

Step 4

When picking constraint subspaces for placing stiffness-changing rods, we favored designs that require fewer rods and rotational symmetry on the three planes. I.e., the two stages should have identical shapes. In particular, rotational symmetry reduces design complexity as both stages share the same geometry. The configurations to enable each mode were also identical along the three axes. Moreover, we also intended to avoid flexure cluttering, which complicates the device's assembly and could cause the flexures to collide.

We started by picking the subspaces with the highest cardinality (Fig. S15c). Yet, as noted, the subspaces shared by the three translations lacked a directional component and were invalid. Therefore, we applied the subspace replacement rule described in eq. 27 and divided the invalid subspaces into two with a lower cardinality (Fig. S15d). This action led to redundancies in selected

constraint spaces: the cardinality-5 constraint subspaces were strict subsets of the newly selected ones (cardinality of 4), as they were shared by fewer modes (Fig. S15e). Therefore, we removed the cardinality-5 subspace that was redundant.

Next, we substituted the rest of the cardinality-5 subspaces with eq. 28 to maintain symmetry. However, the subspace highlighted in Figure S15f presents an undesired option as it may lead to flexure cluttering. I.e., requiring two flexures to pass through the spatial origin. Therefore, we selected two subspaces with a cardinality of three to satisfy the design (Fig. S15f). Finally, an additional constraint subspace was selected to maintain symmetry along all three planes (Fig. S15g), leading to the Venn diagram in Figure S15h.

Steps 5 and 6

The constraint subspaces selected in the previous step led to an identical layout on the three spatial planes passing through the spatial origin. Flexures were added to the design following the requirements stated in Sections 2.3 and 2.7. On each plane, the stiffness-changing flexural rods' extended axes formed a triangle at the compressive side of motions (Fig. S15i). All rods were OD 2 mm and 40 mm in length. No redundant flexures were added to this design.

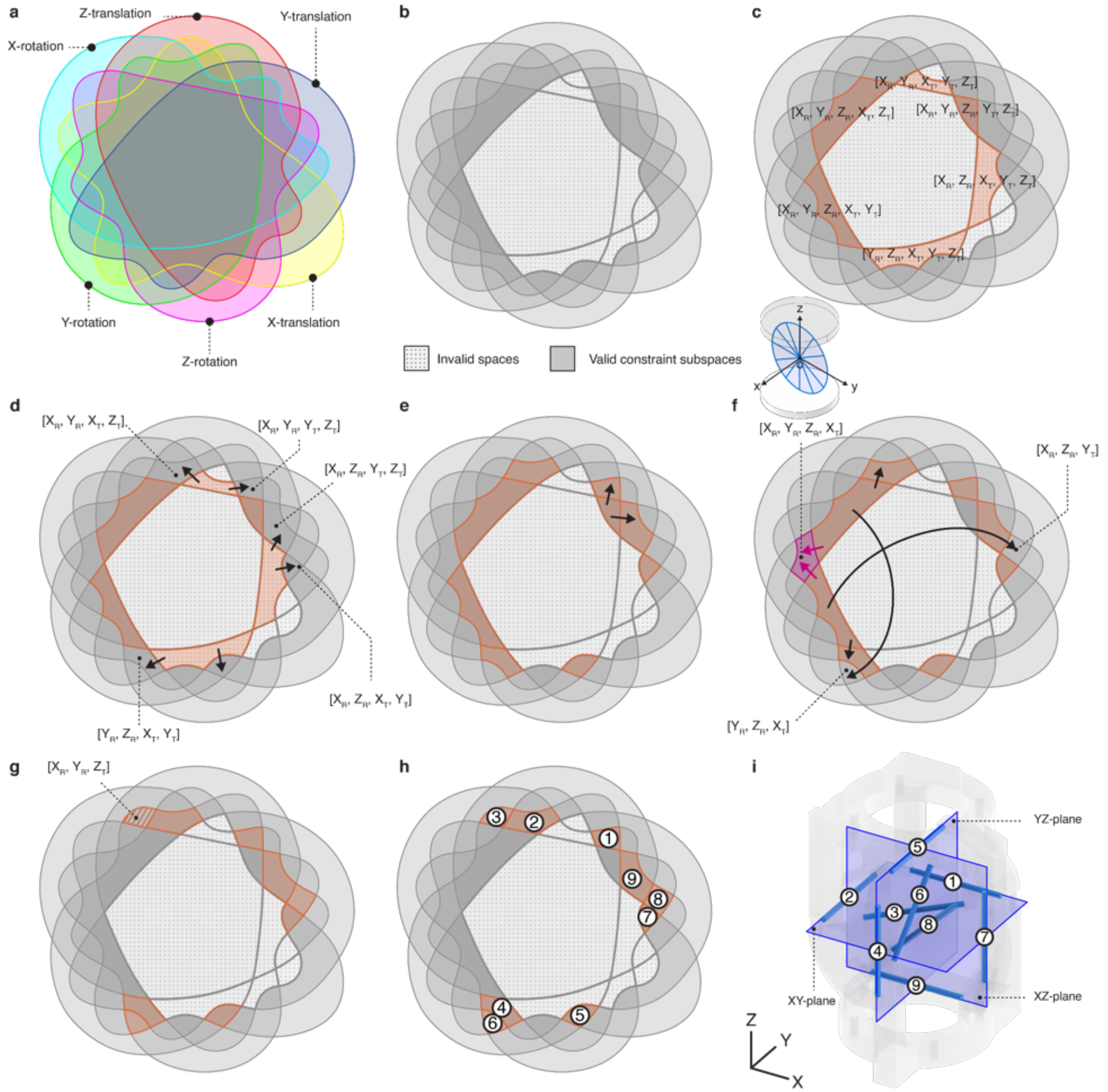

***Figure S15. The constraint subspace selection process shown in Venn diagram representations. a**, The Venn diagram with labeled constituent constraint spaces. **b**, The Venn diagram with colors removed and showing subspaces validity. **c**, Initial constraint subspace selection - selecting those with the highest cardinality. **d**, Constraint subspace substitution to avoid invalid ones. **e**, Constraint subspaces substitution to remove redundancy. **f**, Constraint subspace substitution to avoid cluttering. The option that leads to further cluttering is highlighted in magenta and visualized. **g**, Constraint subspace selection to maintain symmetry. **h**, Placing flexural rods to satisfy constraint subspaces. **i**, Final flexural rod layout. The square brackets indicate the kinematic modes intersected into the marked subspace.*

Supplementary Table S4. 6-DOF device constraint subspace table.

Degree	Is valid	X_R	Y_R	Z_R	X_T	Y_T	Z_T	Space (i.e., $[W]^T$)
1	Y	Y	N	N	N	N	N	[[0, 1, 0, 0, 0, 0]<br>[0, 0, 1, 0, 0, 0]<br>[1, 0, 0, 0, 0, 0]<br>[0, 0, 0, 0, 1, 0]<br>[0, 0, 0, 0, 0, 1]]
1	Y	N	Y	N	N	N	N	[[0, 1, 0, 0, 0, 0]<br>[0, 0, 1, 0, 0, 0]<br>[0, 0, 0, 1, 0, 0]<br>[1, 0, 0, 0, 0, 0]<br>[0, 0, 0, 0, 0, 1]]
1	Y	N	N	Y	N	N	N	[[0, 1, 0, 0, 0, 0]<br>[0, 0, 1, 0, 0, 0]<br>[0, 0, 0, 1, 0, 0]<br>[0, 0, 0, 0, 1, 0]<br>[1, 0, 0, 0, 0, 0]]
1	Y	N	N	N	Y	N	N	[[0, 1, 0, 0, 0, 0]<br>[0, 0, 1, 0, 0, 0]<br>[0, 0, 0, 1, 0, 0]<br>[0, 0, 0, 0, 1, 0]<br>[0, 0, 0, 0, 0, 1]]
1	Y	N	N	N	N	Y	N	[[1, 0, 0, 0, 0, 0]<br>[0, 0, 1, 0, 0, 0]<br>[0, 0, 0, 1, 0, 0]<br>[0, 0, 0, 0, 1, 0]<br>[0, 0, 0, 0, 0, 1]]
1	Y	N	N	N	N	N	Y	[[0, 1, 0, 0, 0, 0]<br>[1, 0, 0, 0, 0, 0]<br>[0, 0, 0, 1, 0, 0]<br>[0, 0, 0, 0, 1, 0]<br>[0, 0, 0, 0, 0, 1]]
2	Y	Y	Y	N	N	N	N	[[0, 0, 1, 0, 0, 0]<br>[1, 0, 0, 0, 0, 0]<br>[0, 1, 0, 0, 0, 0]

								[0, 0, 0, 0, 0, 1]]
2	Y	Y	N	Y	N	N	N	[[0, 0, 1, 0, 0, 0]<br>[1, 0, 0, 0, 0, 0]<br>[0, 0, 0, 0, 1, 0]<br>[0, 1, 0, 0, 0, 0]]
2	Y	Y	N	N	Y	N	N	[[0, 0, 1, 0, 0, 0]<br>[0, 1, 0, 0, 0, 0]<br>[0, 0, 0, 0, 1, 0]<br>[0, 0, 0, 0, 0, 1]]
2	Y	Y	N	N	N	Y	N	[[0, 0, 1, 0, 0, 0]<br>[1, 0, 0, 0, 0, 0]<br>[0, 0, 0, 0, 1, 0]<br>[0, 0, 0, 0, 0, 1]]
2	Y	Y	N	N	N	N	Y	[[0, 1, 0, 0, 0, 0]<br>[1, 0, 0, 0, 0, 0]<br>[0, 0, 0, 0, 1, 0]<br>[0, 0, 0, 0, 0, 1]]
2	Y	N	Y	Y	N	N	N	[[0, 0, 1, 0, 0, 0]<br>[0, 0, 0, 1, 0, 0]<br>[1, 0, 0, 0, 0, 0]<br>[0, 1, 0, 0, 0, 0]]
2	Y	N	Y	N	Y	N	N	[[0, 0, 1, 0, 0, 0]<br>[0, 0, 0, 1, 0, 0]<br>[0, 1, 0, 0, 0, 0]<br>[0, 0, 0, 0, 0, 1]]
2	Y	N	Y	N	N	Y	N	[[0, 0, 1, 0, 0, 0]<br>[0, 0, 0, 1, 0, 0]<br>[1, 0, 0, 0, 0, 0]<br>[0, 0, 0, 0, 0, 1]]
2	Y	N	Y	N	N	N	Y	[[0, 1, 0, 0, 0, 0]<br>[0, 0, 0, 1, 0, 0]<br>[1, 0, 0, 0, 0, 0]<br>[0, 0, 0, 0, 0, 1]]

2	Y	N	N	Y	Y	N	N	[[0, 0, 1, 0, 0, 0]<br>[0, 0, 0, 1, 0, 0]<br>[0, 0, 0, 0, 1, 0]<br>[0, 1, 0, 0, 0, 0]]
2	Y	N	N	Y	N	Y	N	[[0, 0, 1, 0, 0, 0]<br>[0, 0, 0, 1, 0, 0]<br>[0, 0, 0, 0, 1, 0]<br>[1, 0, 0, 0, 0, 0]]
2	Y	N	N	Y	N	N	Y	[[0, 1, 0, 0, 0, 0]<br>[0, 0, 0, 1, 0, 0]<br>[0, 0, 0, 0, 1, 0]<br>[1, 0, 0, 0, 0, 0]]
2	Y	N	N	N	Y	Y	N	[[0, 0, 1, 0, 0, 0]<br>[0, 0, 0, 1, 0, 0]<br>[0, 0, 0, 0, 1, 0]<br>[0, 0, 0, 0, 0, 1]]
2	Y	N	N	N	Y	N	Y	[[0, 1, 0, 0, 0, 0]<br>[0, 0, 0, 1, 0, 0]<br>[0, 0, 0, 0, 1, 0]<br>[0, 0, 0, 0, 0, 1]]
2	Y	N	N	N	N	Y	Y	[[1, 0, 0, 0, 0, 0]<br>[0, 0, 0, 1, 0, 0]<br>[0, 0, 0, 0, 1, 0]<br>[0, 0, 0, 0, 0, 1]]
3	Y	Y	Y	Y	N	N	N	[[1, 0, 0, 0, 0, 0]<br>[0, 1, 0, 0, 0, 0]<br>[0, 0, 1, 0, 0, 0]]
3	Y	Y	Y	N	Y	N	N	[[0, 0, 1, 0, 0, 0]<br>[0, 1, 0, 0, 0, 0]<br>[0, 0, 0, 0, 0, 1]]
3	Y	Y	Y	N	N	Y	N	[[1, 0, 0, 0, 0, 0]<br>[0, 0, 1, 0, 0, 0]<br>[0, 0, 0, 0, 0, 1]]

3	Y	Y	Y	N	N	N	Y	[[1, 0, 0, 0, 0, 0]<br>[0, 1, 0, 0, 0, 0]<br>[0, 0, 0, 0, 0, 1]]
3	Y	Y	N	Y	Y	N	N	[[0, 0, 1, 0, 0, 0]<br>[0, 0, 0, 0, 1, 0]<br>[0, 1, 0, 0, 0, 0]]
3	Y	Y	N	Y	N	Y	N	[[1, 0, 0, 0, 0, 0]<br>[0, 0, 0, 0, 1, 0]<br>[0, 0, 1, 0, 0, 0]]
3	Y	Y	N	Y	N	N	Y	[[1, 0, 0, 0, 0, 0]<br>[0, 0, 0, 0, 1, 0]<br>[0, 1, 0, 0, 0, 0]]
3	Y	Y	N	N	Y	Y	N	[[0, 0, 1, 0, 0, 0]<br>[0, 0, 0, 0, 1, 0]<br>[0, 0, 0, 0, 0, 1]]
3	Y	Y	N	N	Y	N	Y	[[0, 1, 0, 0, 0, 0]<br>[0, 0, 0, 0, 1, 0]<br>[0, 0, 0, 0, 0, 1]]
3	Y	Y	N	N	N	Y	Y	[[1, 0, 0, 0, 0, 0]<br>[0, 0, 0, 0, 1, 0]<br>[0, 0, 0, 0, 0, 1]]
3	Y	N	Y	Y	Y	N	N	[[0, 0, 0, 1, 0, 0]<br>[0, 0, 1, 0, 0, 0]<br>[0, 1, 0, 0, 0, 0]]
3	Y	N	Y	Y	N	Y	N	[[0, 0, 0, 1, 0, 0]<br>[1, 0, 0, 0, 0, 0]<br>[0, 0, 1, 0, 0, 0]]
3	Y	N	Y	Y	N	N	Y	[[0, 0, 0, 1, 0, 0]<br>[1, 0, 0, 0, 0, 0]<br>[0, 1, 0, 0, 0, 0]]
3	Y	N	Y	N	Y	Y	N	[[0, 0, 0, 1, 0, 0]<br>[0, 0, 1, 0, 0, 0]<br>[0, 0, 0, 0, 0, 1]]

3	Y	N	Y	N	Y	N	Y	[[0, 0, 0, 1, 0, 0]<br>[0, 1, 0, 0, 0, 0]<br>[0, 0, 0, 0, 0, 1]]
3	Y	N	Y	N	N	Y	Y	[[0, 0, 0, 1, 0, 0]<br>[1, 0, 0, 0, 0, 0]<br>[0, 0, 0, 0, 0, 1]]
3	Y	N	N	Y	Y	Y	N	[[0, 0, 0, 1, 0, 0]<br>[0, 0, 0, 0, 1, 0]<br>[0, 0, 1, 0, 0, 0]]
3	Y	N	N	Y	Y	N	Y	[[0, 0, 0, 1, 0, 0]<br>[0, 0, 0, 0, 1, 0]<br>[0, 1, 0, 0, 0, 0]]
3	Y	N	N	Y	N	Y	Y	[[0, 0, 0, 1, 0, 0]<br>[0, 0, 0, 0, 1, 0]<br>[1, 0, 0, 0, 0, 0]]
3	N	N	N	N	Y	Y	Y	[[0, 0, 0, 1, 0, 0]<br>[0, 0, 0, 0, 1, 0]<br>[0, 0, 0, 0, 0, 1]]
4	Y	Y	Y	Y	Y	N	N	[[0, 1, 0, 0, 0, 0]<br>[0, 0, 1, 0, 0, 0]]
4	Y	Y	Y	Y	N	Y	N	[[1, 0, 0, 0, 0, 0]<br>[0, 0, 1, 0, 0, 0]]
4	Y	Y	Y	Y	N	N	Y	[[0, 1, 0, 0, 0, 0]<br>[1, 0, 0, 0, 0, 0]]
4	Y	Y	Y	N	Y	Y	N	[[0, 0, 1, 0, 0, 0]<br>[0, 0, 0, 0, 0, 1]]
4	Y	Y	Y	N	Y	N	Y	[[0, 1, 0, 0, 0, 0]<br>[0, 0, 0, 0, 0, 1]]
4	Y	Y	Y	N	N	Y	Y	[[1, 0, 0, 0, 0, 0]<br>[0, 0, 0, 0, 0, 1]]
4	Y	Y	N	Y	Y	Y	N	[[0, 0, 0, 0, 1, 0]

								[0, 0, 1, 0, 0, 0]]
4	Y	Y	N	Y	Y	N	Y	[[0, 0, 0, 0, 1, 0]<br>[0, 1, 0, 0, 0, 0]]
4	Y	Y	N	Y	N	Y	Y	[[0, 0, 0, 0, 1, 0]<br>[1, 0, 0, 0, 0, 0]]
4	N	Y	N	N	Y	Y	Y	[[0, 0, 0, 0, 1, 0]<br>[0, 0, 0, 0, 0, 1]]
4	Y	N	Y	Y	Y	Y	N	[[0, 0, 1, 0, 0, 0]<br>[0, 0, 0, 1, 0, 0]]
4	Y	N	Y	Y	Y	N	Y	[[0, 0, 0, 1, 0, 0]<br>[0, 1, 0, 0, 0, 0]]
4	Y	N	Y	Y	N	Y	Y	[[1, 0, 0, 0, 0, 0]<br>[0, 0, 0, 1, 0, 0]]
4	N	N	Y	N	Y	Y	Y	[[0, 0, 0, 1, 0, 0]<br>[0, 0, 0, 0, 0, 1]]
4	N	N	N	Y	Y	Y	Y	[[0, 0, 0, 0, 1, 0]<br>[0, 0, 0, 1, 0, 0]]
5	Y	Y	Y	Y	Y	Y	N	[[0, 0, 1, 0, 0, 0]]
5	Y	Y	Y	Y	Y	N	Y	[[0, 1, 0, 0, 0, 0]]
5	Y	Y	Y	Y	N	Y	Y	[[1, 0, 0, 0, 0, 0]]
5	N	Y	Y	N	Y	Y	Y	[[0, 0, 0, 0, 0, 1]]
5	N	Y	N	Y	Y	Y	Y	[[0, 0, 0, 0, 1, 0]]
5	N	N	Y	Y	Y	Y	Y	[[0, 0, 0, 1, 0, 0]]
6	N	Y	Y	Y	Y	Y	Y	[]

4.2 Device Design

The fixed and mobile stages were designed after placing the rods and had identical shapes due to the flexures' rotational symmetry (Fig. S16a). The models had pre-defined bolt holes (3mm diameter M3 screw) at the base to connect to mechanical testing jigs, and structural arms extended from the base and met the stiffness-changing rods at the through holes housing them. All through holes had a depth of 5 mm. We added grooves to house the conductive wires connecting the rods' resistive heating wires. Specifically, the grooves had a diameter (0.5 mm) matching the 26-gauge conductive wires, and the wires were glued to the stages to prevent delamination.

The stages were printed using the FDM printer as three separate parts (Fig. S16b). We oriented the parts to ensure the layering direction was perpendicular to the length of the structural arms, which helped avoid layer delamination when the devices were loaded.

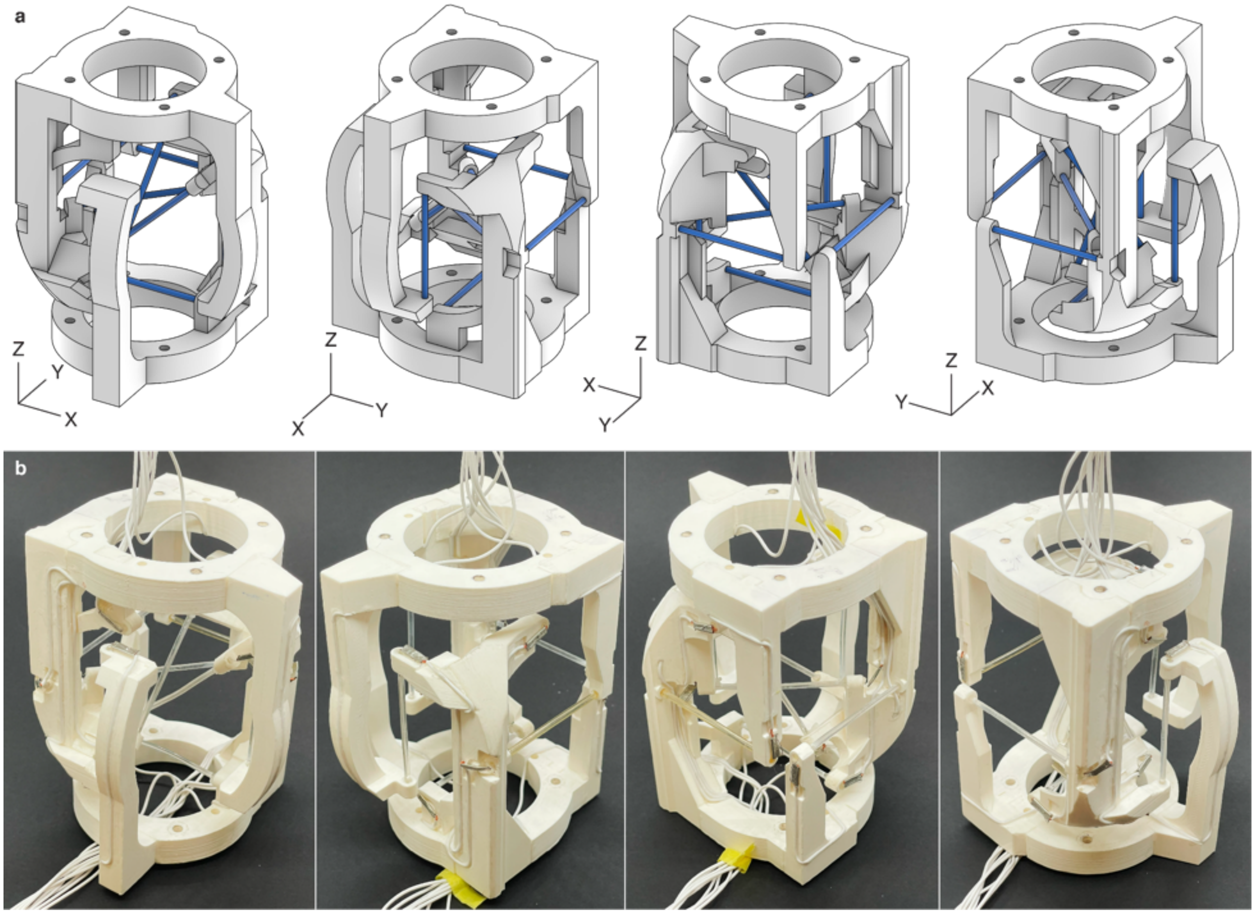

Figure S16. The 6-DOF device design. a*, The device design without mechanical features rendered in different view angles.* ***b****, The final, assembled device shown in angles corresponding to subfigure a. Scale bar, 20 mm.*

We note that it is possible to use the rational design algorithm to produce varying designs for a given kinematic reconfiguration. Figure S17 shows an alternate 6-DOF device design with a different flexure layout. The algorithm solves for viable and needed constraint subspaces and flexure placements, and mechanism designers may interpret its output and employ different heuristics in modeling the flexural elements (as noted in Supplementary Note 2). A targeted reconfigurable mechanism may be satisfied by multiple constraint subspace combinations. Compared with the device shown in Figure 3, the device in Figure S17 requires more rods to create the required reconfiguration layout, and the rods are more cluttered.

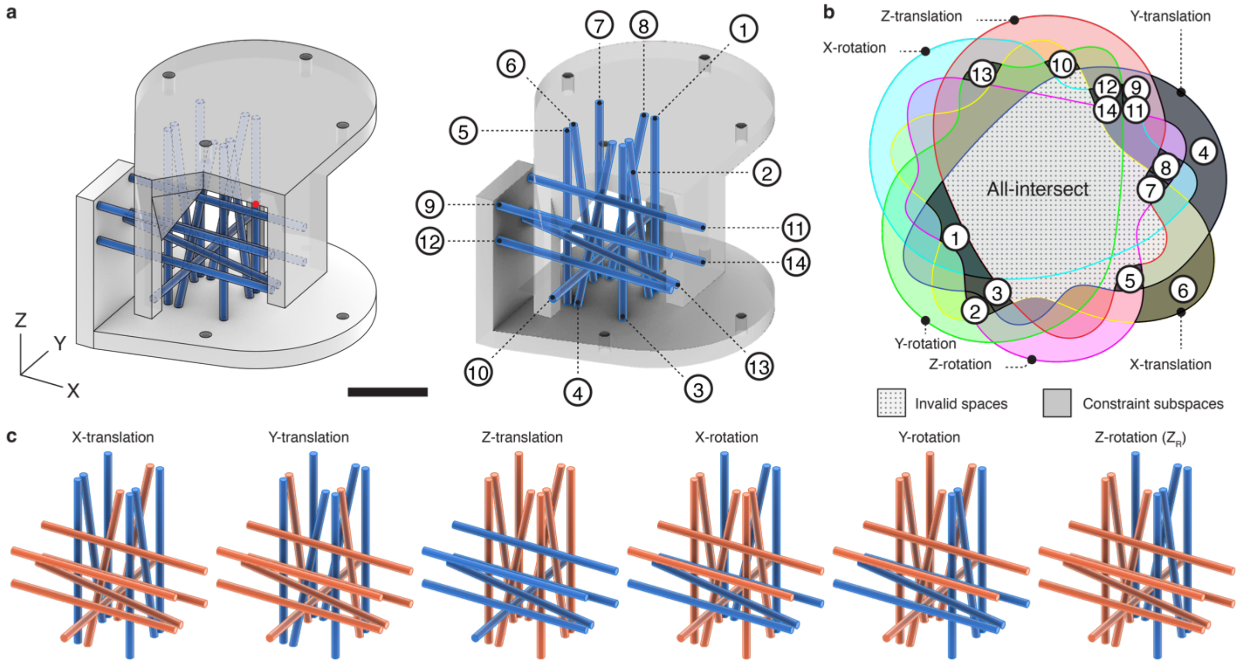

***Figure S17. Alternative 6-DOF device design. a**, Device design and reconfigurable flexure rod layout. The red dot shows the pivot center. Scale bar, 20 mm. **b**, The Venn diagram showing each rod's constraint subspaces membership. **c**, Illustrations of the device's flexure configuration to enable each DOF (orange: heated, blue: cold).*

Supplementary Note 5. Wearable Haptic Device Design and Iteration

5.1 Design Process Summary

The wearable devices were targeted at an application scenario where the kinematics, stiffness, and motional resistance are essential to the device's performance. Therefore, the algorithm, FE simulation, and the analytical stiffness model were used to iterate the design. At a high level, we started by setting the design criteria based on human perception and physiological literature. Next, we took the kinematic specifications and applied the rational design pipeline to identify flexure placements (i.e., constraint subspaces) required for each joint. Lastly, we used FE simulations to evaluate the design's performance and the analytical stiffness model to strategize design modifications (i.e., flexure geometry parameters and adding redundant flexures). Specifically, we iterated between FE simulation and modifying flexure parameters until the targeted performance was satisfied. In short, the three steps to design a reconfigurable joint were

Step 1: Defining design criteria based on human perception and physiology.

Step 2: Identify flexure placements using the multimodal kinematics design algorithm.

Step 3: Iterate designs using FE simulation and the analytical stiffness model until the targeted performance is reached.

5.2 Arm-wearable: Design Criteria

This design was aimed to create a device worn on the human forearm, wrist, and finger to provide individual DOF locking and locking depending on the application context (Video S2-S3). Table S5 summarizes human perceptual and physiological performances at each of the joints. We chose these joints as they are located at the are the most prominent joints associated with kinesthetic perception[18,19].

We based our arm torque specifications on Gupta et al.[20] The torques at each joint were set as a fraction of the human isometric strength. In particular, forearm supination and wrist rotations were set at 50% and 25% of a healthy adult's isometric strength. On the other hand, the finger joints' isometric strength was based on Milner et al.[18] and directly used as the device design criteria. Finally, the proprioception rotational just noticeable difference (JND) was based on Reissner et al.[19]

Supplementary Table S5. The arm-wearable device design criteria.

Joint	Isometric Strength (Nm)[18,21,22]	Design criteria (Nm)[18,20]	Just noticeable difference (degree)[19]
Forearm	10	5	8
Wrist flexion	10.92	4	6
Wrist deviation	8.46	4	7
Metacarpophalangeal (MP)	0.8	0.8	8
Proximal interphalangeal (PIP)	0.37	0.37	7
Distal interphalangeal (DIP)	0.14	0.14	9

5.3 Arm-wearable: Rational Design of Flexure Placements

The arm-wearable device contained five joints: forearm pronation, wrist flexion and deviation, metacarpophalangeal (MP), proximal interphalangeal (PIP), and distal interphalangeal (DIP) joints. We designed the joints individually and combined them in series to form the final device. To design the arm-wearable device, we took measurements of the wearer and modeled the DOF axes in 3D model software. Next, placeholder geometries (i.e., plain shells) were placed at the opposite end of the joints, and the spaces between them were designated for placing flexures.

Forearm and finger joints (1 DOF rotation)

The forearm and finger joints have the same kinematic DOF, albeit with a different orientation. The forearm's rotation axis runs along the length of the forearm, whereas the finger joints' rotation axes run perpendicular to the finger. Still, we designed these joints to have two kinematic modes - locked (0DOF) and unlocked (1DOF of rotation). The constraint subspaces calculated by the algorithm are shown in Figure S18 as a Venn diagram. It is worth noting that the shared constraint subspace was the same as the unlocked mode; thus, no stiffness-changing flexures were needed to instate the unlocked mode. On the other hand, at least one stiffness-changing flexure was needed to instate the locked mode with 0DOF. Precisely, the stiffness-changing flexure's extension line must not coincide with nor be parallel to the rotation axis.

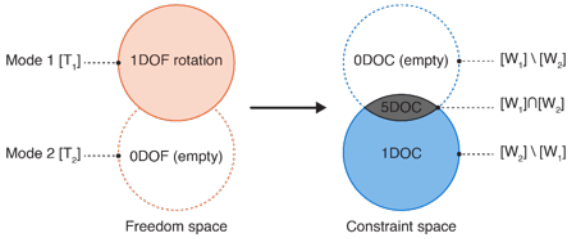

Figure S18. Arm-wearable device kinematic mode specification and Venn diagram (1DOF rotation). *This specification was applied to the forearm and finger joints design.*

Wrist joint (2 DOF rotation)

The wrist joint comprises two rotation axes perpendicular to the length of the arm. Wrist flexion-extension denotes rotations where the palm's trajectory is perpendicular to itself, whereas the wrist's ulnar deviation denotes rotations where the palm's trajectory is in plane with itself. We defined two kinematic modes as input, each enabling a rotation axis. The resulting constraint subspaces are shown in the Venn diagram provided in Figure S19. We note that in this design, in addition to the two constraint modes, it was also possible to turn both rotations on or off simultaneously by softening or stiffening all stiffness-changing flexures, respectively. Therefore, the device was capable of a total of four kinematic modes.

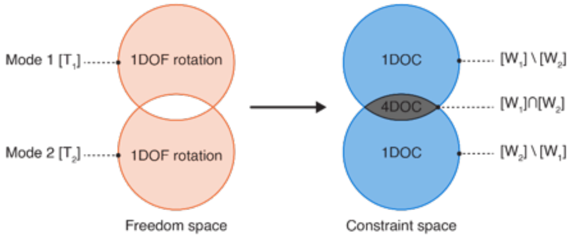

Figure S19. Arm-wearable device kinematic mode specification and Venn diagram (2DOF rotation). *This specification was applied to the wrist joint design.*

5.4 Arm-wearable: Flexure Design Iterations

After identifying the flexure placements using the algorithm, we replaced the shared constraint subspace with the skeletal joint. The skeletal joint readily and exactly constrained the joint to have the fully unlocked DOF. Still, additional passive flexures were added to the forearm and finger joints to maintain the spacing between the two stages. We note that in these device designs, there was no need nor freedom to select different subsets of constraint subspaces since the ones resulting from the algorithm were all viable and efficient. Nevertheless, we added non-redundant flexures to each device (V1 in Fig. S20, S22, and S24) to initiate design iteration toward the design criteria.

Forearm joint

The forearm pronation joint design iteration (Fig. S20) began with a stiffness-changing flexural rod between the stages (V1), which yielded a much lower buckling plateau than the expected load (5 Nm) in the FE simulation. We then added more flexures (V2) by rotating and copying the stiffness-changing flexure twelve times about the forearm pronation axis. Yet, the stiffness was still low as the rods were somewhat oriented parallel to the rotation axis (i.e., θ was too small). Thus, we oriented the flexures to become more perpendicular to the rotation axis to arrive at the final design (V3 and Fig. S21).

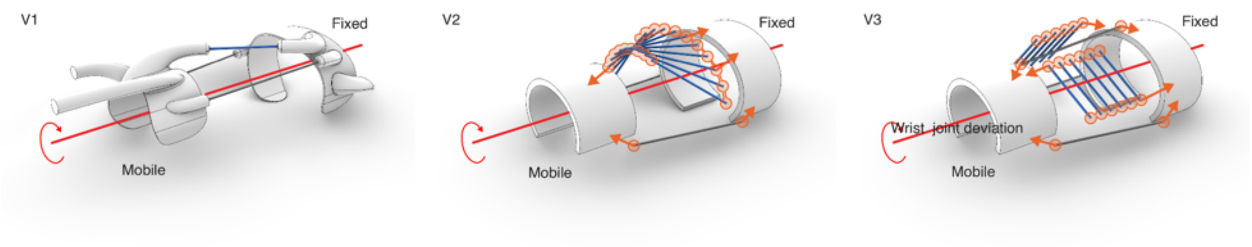

Figure S20. The forearm joint's design iteration. *The red axis marks the kinematic DOF (forearm pronation). The orange marks and arrows indicate the remote rigid connection applied in FE simulations.*

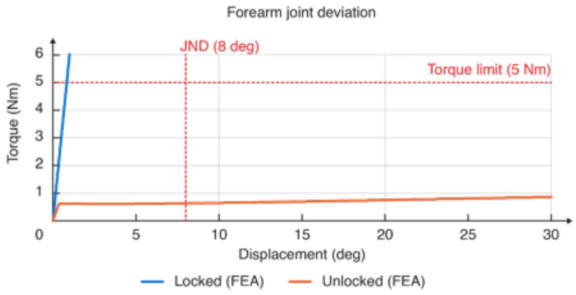

Figure S21. The final forearm joint design performance evaluation using FE simulation.

Finger joints

The finger joints shared an identical layout (Fig. S22). Both passive and stiffness-changing flexural rods were added at an angle with respect to the axis to avoid cluttering around their endpoints, and a passive flexure was added between the stages to restrain the two stages' relative position (Base). Since each joint had a different max torque requirement, we modulated the stiffness-changing rods' distance from the rotation axis to adjust their resistance against the motion, arriving at the final design. Due to the low torque limit at the finger joints, the passive flexures had a reduced diameter of 1.5 mm. Finally, the MP joint's torque limit is 2.35 times higher than the others, and we mirrored

and copied the stiffness-changing flexure about the rotation center to create a design with higher stiffness (Fig. S23).

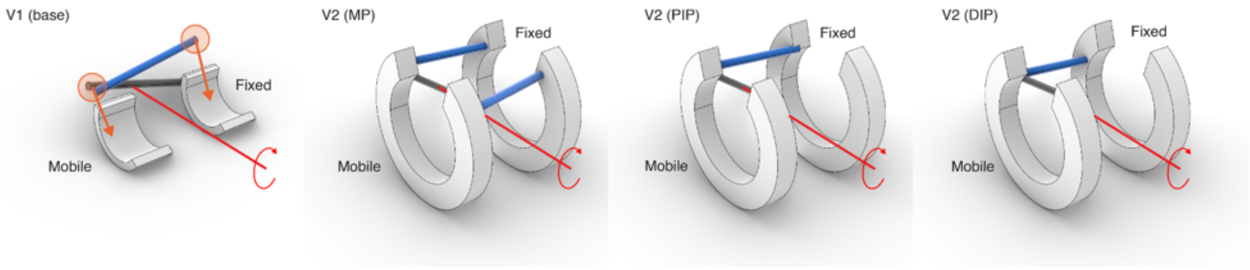

Figure S22. The finger joints' design iteration. *The red axis marks the kinematic DOF. The orange marks and arrows indicate the remote rigid connection applied in FE simulations.*

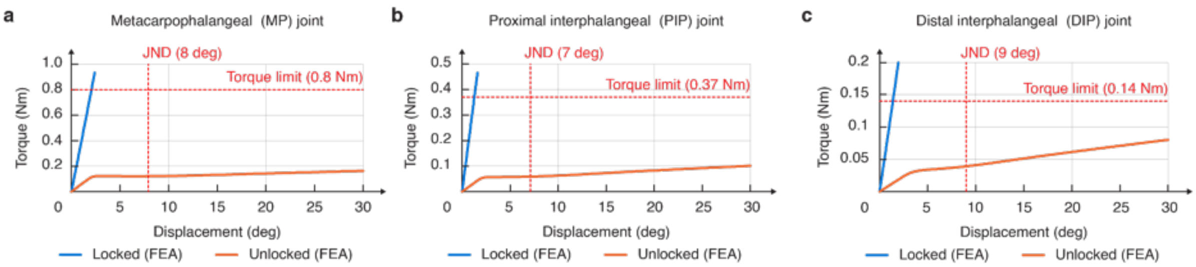

***Figure S23. The final finger joint design performance evaluation using FE simulation**. **a**, the MP joint; **b**, the PIP joint; **c**, the DIP joint.*

Wrist joints

We explored different flexural rod layouts according to the algorithm's output (Fig. S24). No passive flexures were added to this design to avoid cluttering and collision. Several design variations (v1-v4) were explored, but they required long structural arms connecting the stages and rods, which led to increased material usage, weight, and mechanical integration challenges. Consequently, we opted for a design (v5) that required less structural connection. Next, we adjusted the number of rods to ensure the device had effective locking effects against the expected torque (v6, see also Fig. S25).

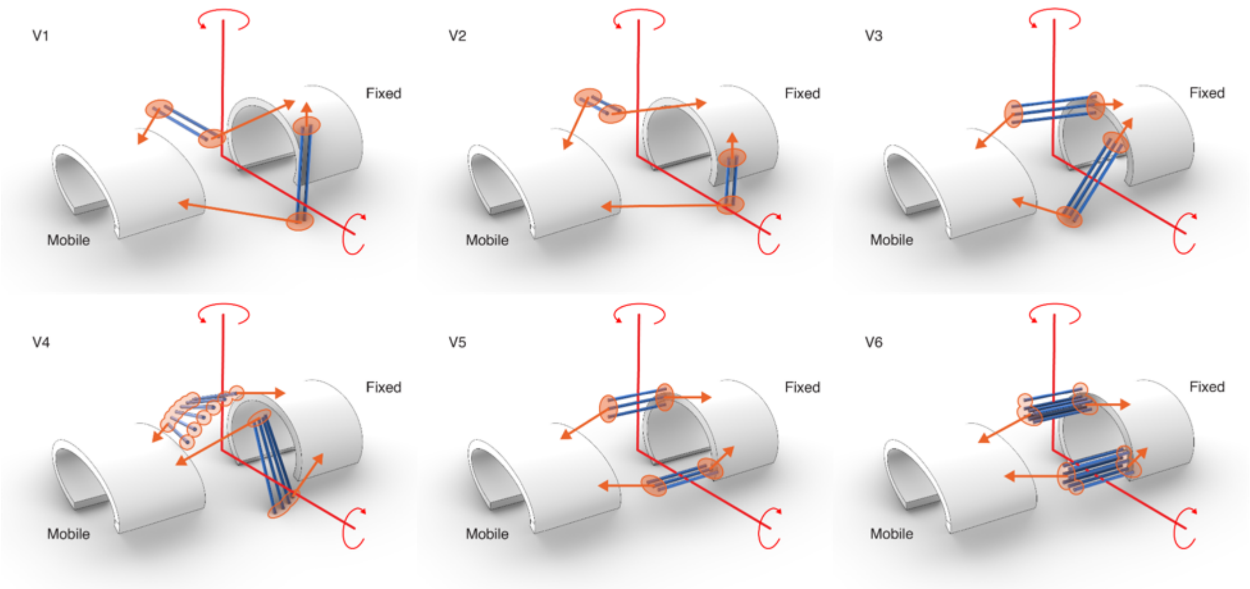

Figure S24. The wrist joint's design iteration. *The red axis marks the kinematic DOF. The orange marks and arrows indicate the remote rigid connection applied in FE simulations.*

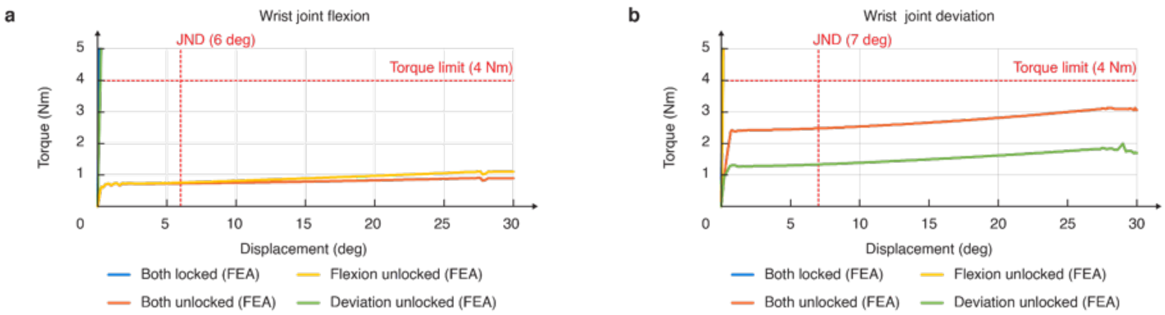

Figure S25. The final wrist joint design performance evaluation using FE simulation. a*, evaluation against wrist flexion.* ***b****, evaluation against wrist deviation.*

5.5 Arm-wearable: Device Design

Once the flexure layout at each joint had been decided, we collated all joints into one file to model the rigid stages (Fig. S26a). Structural arms extending from the stages - modeled as cuffs around the arm - connect and house the flexure endpoints. The stages were modeled to be slightly smaller than the wearer's arm circumference so that the device would gently wrap around and cling to the wearer's body without requiring additional locking mechanisms. FE simulations were used to iterate the rigid stages to minimize their deformation within the joint torque limit. The device was fabricated using the SLS printer. Due to the printer's size constraint, the stages were divided into

smaller parts for printing (Fig. S26). Figure S27-S29 further shows the wearer exercising each joint under their unlocked modes.

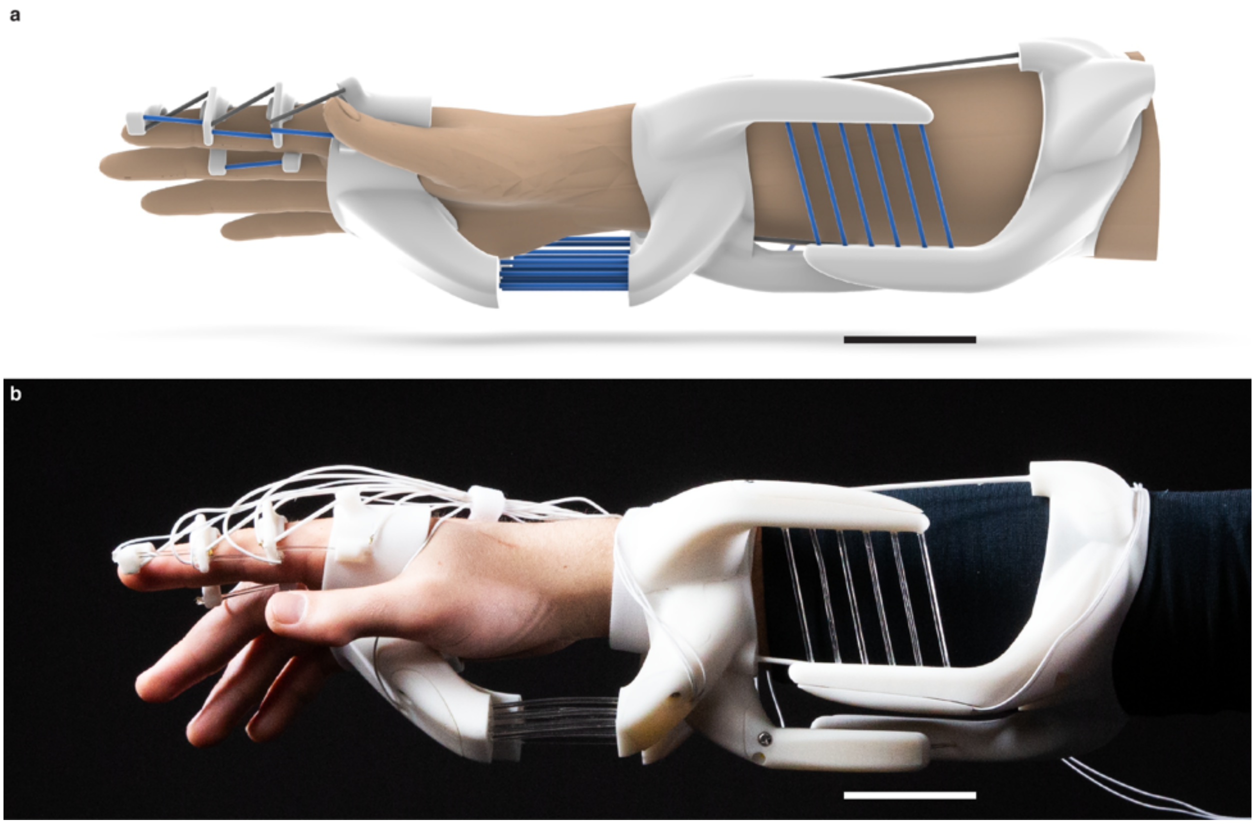

***Figure S26. The arm-wearable device design. a**, A render of the device design without mechanical features. **b**, The final, assembled device. Scale bar, 50 mm.*

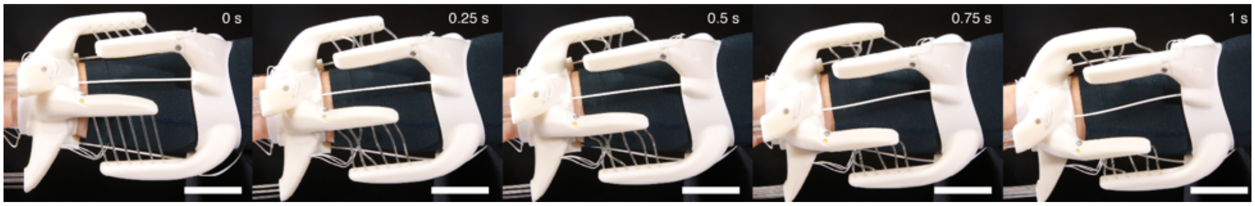

***Figure S27. Pictures of the arm-wearable device's forearm joint in the unlocked state.** Scale bar, 50 mm.*

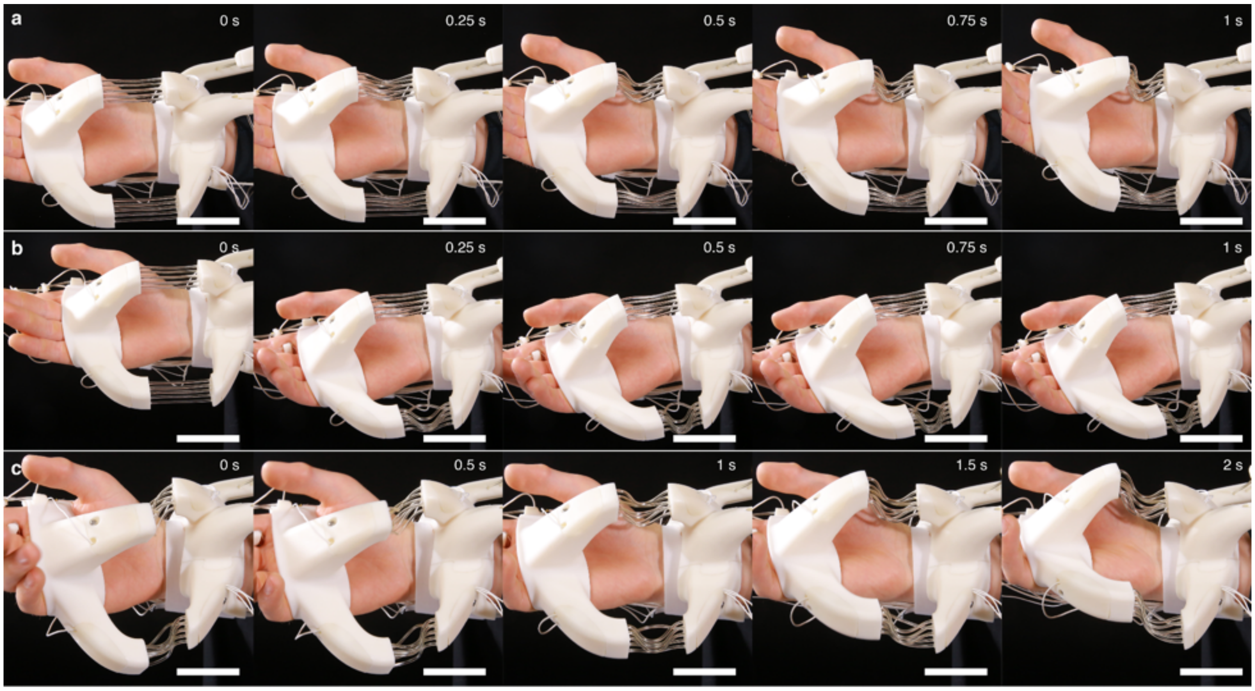

***Figure S28. Pictures of the arm-wearable device's forearm joint in different unlocked modes. a**, The wearer exercising wrist flexion in the flexion-unlocked mode. **b**, The wearer exercising wrist deviation in the deviation-unlocked mode. **c**, The wearer exercising both DOF in the both-DOF-unlocked configuration. Scale bar, 50 mm.*

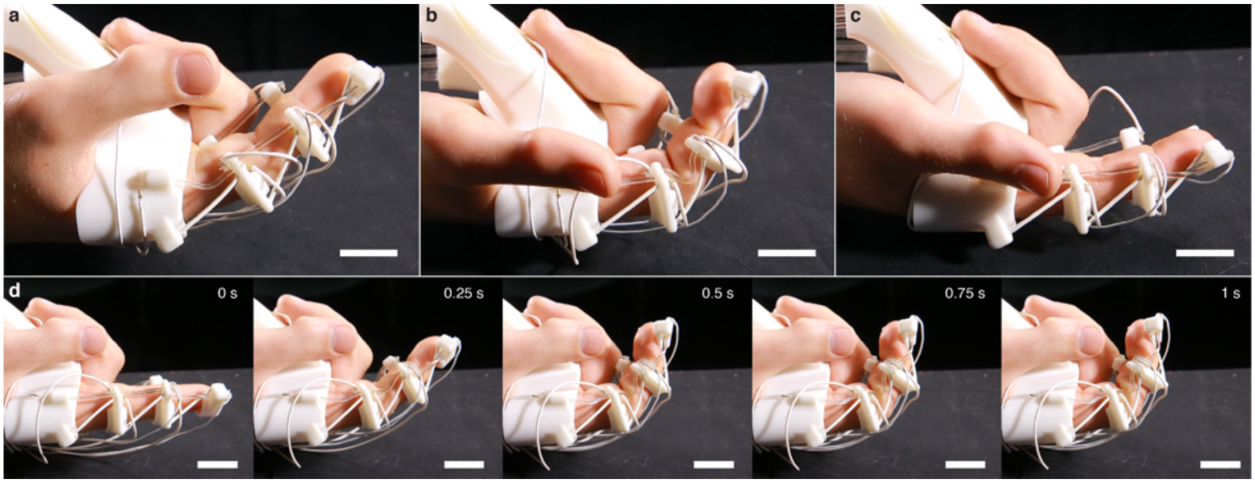

***Figure S29. Pictures of the arm-wearable device's finger joints in different unlocked modes. a**, The wearer exercising MP flexion in the MP-unlocked mode. **b**, The wearer exercising PIP flexion in the PIP-unlocked mode. **c**, The wearer exercising DIP flexion in the DIP-unlocked mode. **d**, The wearer exercising all finger joints with all joints unlocked. Scale bar, 20 mm.*

5.6 Haptic Thimble: Design Criteria

The haptic thimble device was designed to fit the index finger (Video S5), and the design criteria were based on the forces and compliance discrimination of the fingertip in an explorative task (i.e., using fingers to explore the softness of a surface). Zoeller et al.[23] reported that a healthy adult's index finger could exert a compressive force of 25 N in softness explorations, and the just noticeable difference in compliance is 0.05 mmN^{-1}, which converts to a stiffness of 20 Nmm^{-1}. In our design, we assumed the fingertip applied forces over a 10 x 10 mm^2 surface area at the mobile stage, and the flexures were confined in a 20 cubic millimeter space to ensure the device had a small footprint.

5.7 Haptic Thimble: Rational Design of Flexure Placements

The two kinematic modes were defined as one with 6DOF and the other with 0DOF (Fig. S30). This specification led to an empty shared constraint subspace without passive flexures. However, the stiffness-changing flexures should create a full-ranked linear screw space.

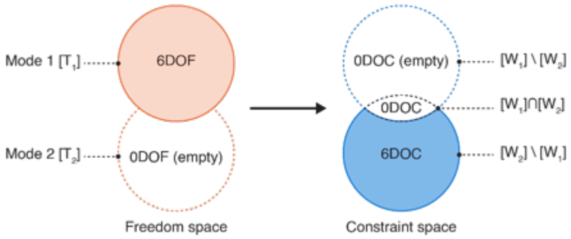

Figure S30. The thimble device's kinematic mode specification and Venn diagram.

5.8 Haptic Thimble: Flexure Design Iterations

Flexural rods (OD 1.5 mm) were added to the 20 cubic millimeter space in mirror symmetry about the central transverse plane. Figure S31 shows the flexural rod layout iterations. We started by adding six flexural rods to the design, yet the stiffness was insufficient to fully lock the device in the stiffest mode under a 25 N load. Therefore, we added more rods in the following design iterations. Note that a pair of flexures with a small θ angle was added to provide high stiffness in the locked state. However, they also caused the flexures to become cluttered and prone to collision when bent (which may cause heater transfer). We arrived at the final design (V6) by spacing out the flexures and replacing the vertical flexure pairs with a single rod located at the central plane with a larger diameter (OD 2 mm) to increase its stiffness against axial displacement loads.

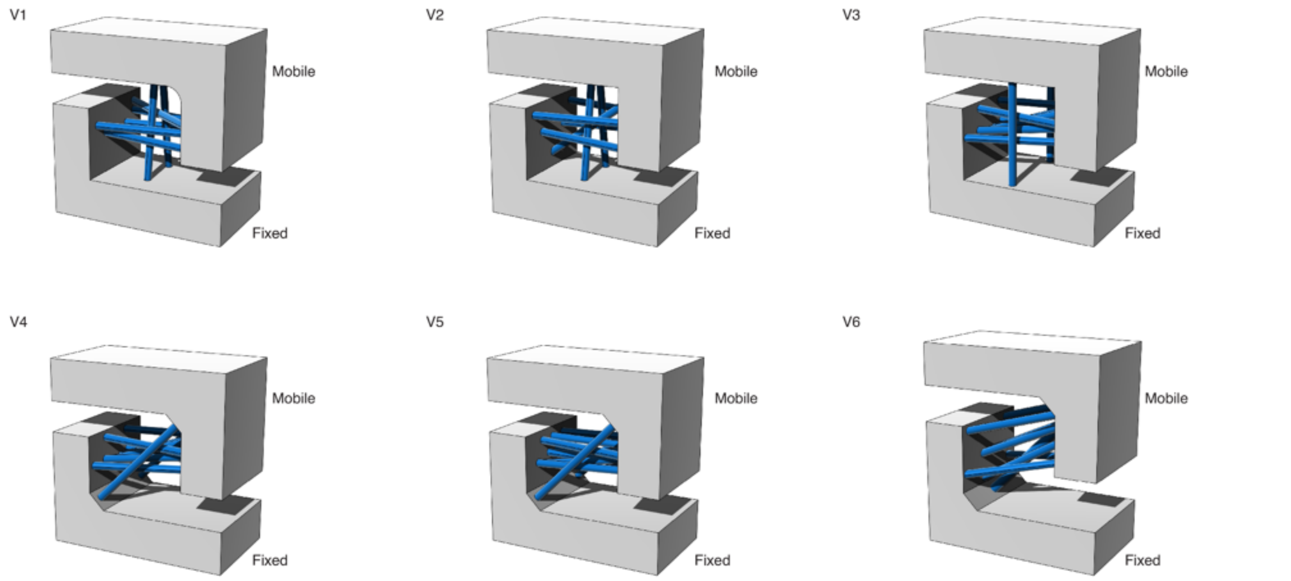

Figure S31. The thimble device's design iteration.

5.9 Haptic Thimble: Device Design

Housing through holes, wiring chambers, and service panels were added to the thimble following finding flexural rod placements (Fig. S32). We added a cylindrical void to house the finger at the mobile stage. The fixed stage was modeled into a smooth, round surface so that the device could come into contact with an external surface at different angles. The device was printed using the SLS printer; both stages are printed as a singular part.

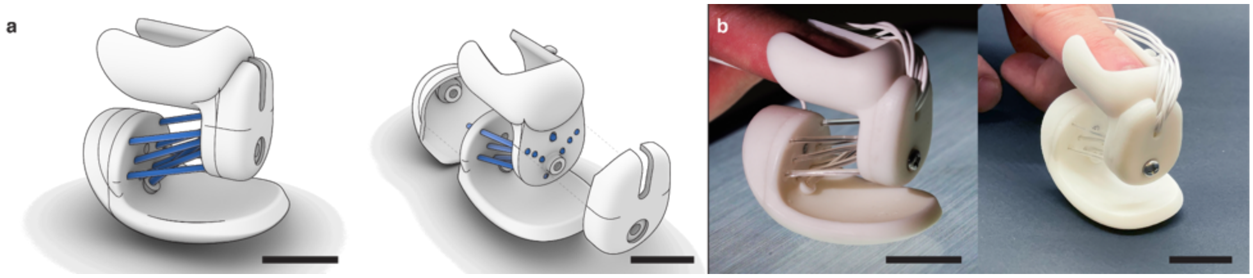

***Figure S32. The Haptic thimble device design. a**, The device digital model rendered from different angles. **b**, The final, assembled device shown in angles corresponding to subfigure **a**. Scale bar, 20 mm.*

Supplementary Note 6: Material Characterization and Mechanical Experiment of Devices

6.1 Dynamic Mechanical Analysis (DMA)

The glass transition temperature (Tg) of the sample (cross-linked epoxy resin with a ratio of 10:4) was determined with a Dynamic Mechanical Analyser (DMA, RSA–G2 Deta; TA Instruments). Samples were heated from room temperature to 80 °C and cooled to 30°C. The sample dimension used for the DMA test was 12.42 x 8.63 x 0.85 mm^3. The observed glass transition temperature (Tg) from the test was 54.0 °C (Fig. S33), where tan δ peaked at 0.571.

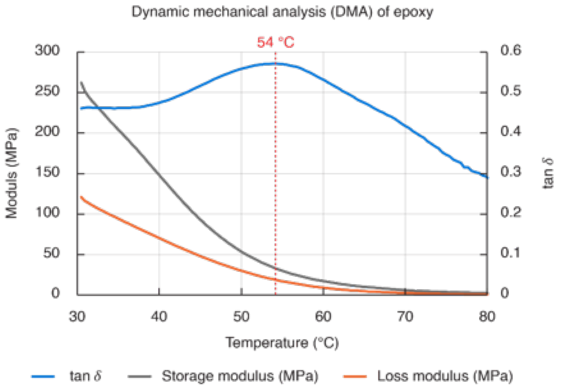

Figure S33. DMA test result for the epoxy sample.

6.2 Material Characterization

Dog bone (ASTM D412) samples were cast with a mixing ratio of 10:4 (monomer: crosslinker) and characterized using mechanical tensile tests under room temperature (RT) and 54°C (Fig. S34). The dog bone samples were heated using hot air from both sides, and their temperature was monitored using a FLIR camera. The strain rate was 10 mm/min. The observed young modulus at room temperature and 54°C were 1135.4 ± 180.7 and 20.86 ± 7.81 MPa, respectively. The strain rate at failure was 11.84 ± 3.94 and 31.75 ± 1.72 % for the RT and 54°C samples, respectively. The Young's modulus and elongation at break changed 54.4 times and 0.37 times for the RT and 54°C samples, respectively.

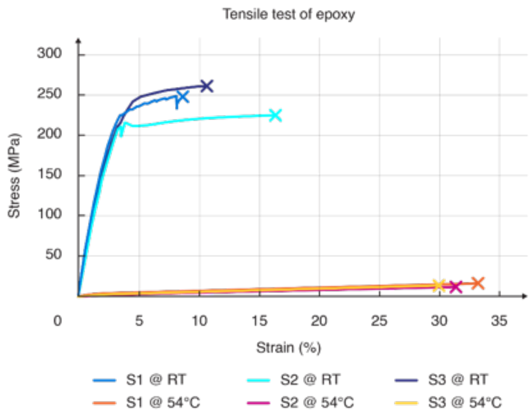

Figure S34. Mechanical properties of Epoxy under room temperature and 54℃*. S stands for samples 1 through 3 for each temperature condition.*

6.3 Flexural Rod Characterization

The flexural rod with heating wire (50 mm length) was subjected to compression at 1% strain (0.5 mm displacement) at a strain rate of 5 mm/min at RT and 54 °C. Both flexure rods with OD 1.5 and 2.0 mm were tested and modeled separately (Fig. S35). The equivalent modulus of elasticity was calculated at 0.1% strain by dividing the load by strain. For rods of OD 2 mm, the equivalent modulus was observed to be 2958.37 ± 50.19 MPa and 152.02 ± 15.74 MPa for the cold and heated states, respectively. For rods of OD 1.5 mm, the equivalent modulus was observed to be 2668.87 ± 1370.85 MPa and 480.85 ± 179.203 MPa for the cold and heated states, respectively.

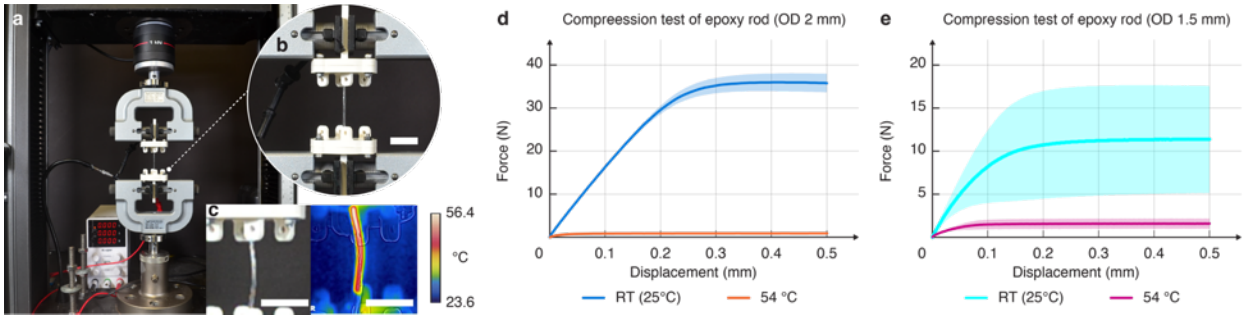

Figure S35. Flexural rod characterization test setup and results. a*, The compression test setup.* ***b****, A zoomed-in image of the sample flexural rod before the test. Scale bar, 20 mm.* ***c****, The heated flexural rod at the maximum load viewed from the camera. The image on the right shows the corresponding thermal image. Scale bar, 20 mm.* ***d****, The load curves of the 2 mm outer diameter rods. Data are means ± s.d. n = 3 samples.* ***e****, The load curves of the 1.5 mm outer diameter rods. Data are means ± s.d. n = 3 samples.*

While the rods are not extensible due to the inclusion of a metallic heating wire, the rods also exhibit a stiffness change when subjected to tensile loads (Fig. S36). Under both RT and 54 °C, a

50 mm long, OD 2 mm rod exhibited a relatively linear load-displacement curve under tension due to the lack of buckling plateaus. The forces required to stretch the rod by 1% strain varied by 6.71 times, changing from 36.60 ± 1.07 N at RT to 5.45 ± 0.94 N at 54 °C. In comparison, the forces required to compress the rod by 1% varied 38.9 times, changing from 35.79 ± 2.14 N at RT to 0.92 ± 0.08 N at 54 °C.

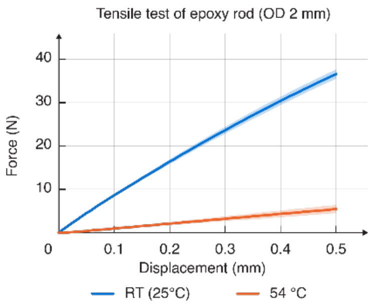

Figure S36. Flexural rod tensile characterization (50 mm long, OD 2 mm). *Data are means ± s.d. n = 3 samples.*

Figure S37 shows a characteristic OD 2 mm rod's hysteresis under cyclic compression. At room temperature, the rod had a 15.67% hysteresis in the first loading cycle, which subsequently dropped to 10.56% in the second and 9.51% in the fifth cycle. Similarly, at 54 °C, the initial loading cycle has the highest hysteresis of 38.33%, which then decreased to 18.64% and 16.91% in the second and fifth loading cycles, respectively.

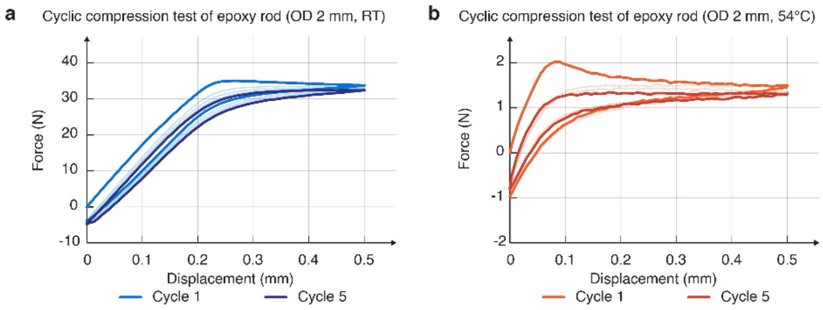

***Figure S37.Characteristic flexural rod hysteresis plots. a**, The rod under room temperature. **b,** The rod under 54℃. The faded-out curves show intermediate cycles between cycle 1 and cycle 5.*

Figure S38 shows a rod's (OD 2 mm, 50 mm long) shape memory effect that allowed it to recover from deformation. When deformed along its degree of freedom (i.e., 10 mm lateral deflection), a rod may recover most of the deformation without external loads. However, when subjected to a

deformation along its degree of constraint (i.e., 5 mm compression), the rod was unable to completely recover from buckling.

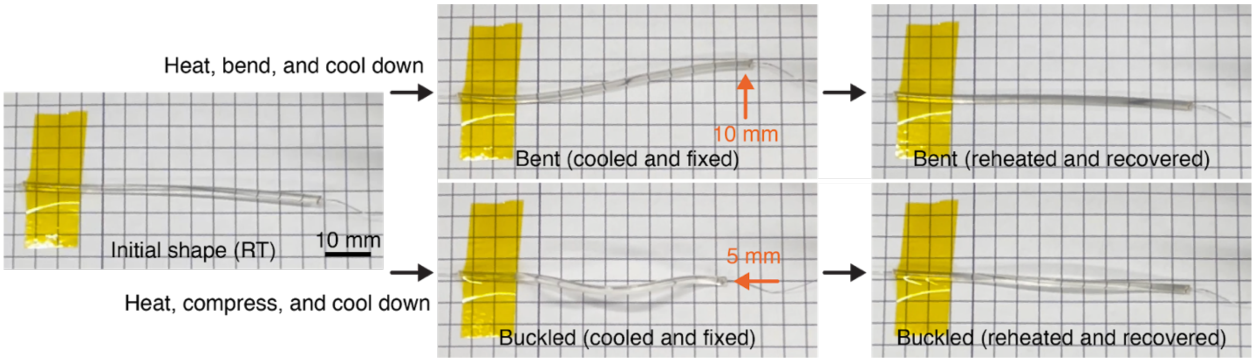

Figure S38. Flexure rod recovery after bending and buckling.

6.4 Mechanical Tests Protocol

The flexural rods and devices were mechanically tested using a Universal Testing Machine (UTM, Instron 5969) equipped with a 1 kN Load cell. All tests were repeated for multiple cycles, and the second load cycles were used in the reported plots unless otherwise specified. The 50 mm long stiffness-changing flexural rods were subjected to a compressive strain of 1% (0.5 mm, displacement) at 5 mm/min for five cycles at cold and hot states.

Three 6DOF devices were fabricated and tested for translations along and rotations (bending) about the X, Y, and Z axis in the locked and unlocked states (Fig. S39). The loads were applied at 10 mm/min for five cycles. For translational motions, the device (Fig, S39a, b) was subjected to a displacement of 1 mm (1% of the device's total length of 100 mm). Since it was difficult to apply the load as a pure toque for XY rotations, we mounted the devices as cantilever beams and subjected lateral loads (2 mm, 2% with respect to device length) to their free end to report their deflection (Fig. S39c). A displacement load of 2 mm (2% with respect to device length) accounted for the compounded displacement (i.e., the free end is allowed to translate and rotate simultaneously). For Z rotations, the load was applied through a loading arm 50 mm away from the rotation axis as a pure torque (Fig. S39d). The displacement load corresponded to 2.29° at a load rate of 11.46°/min.

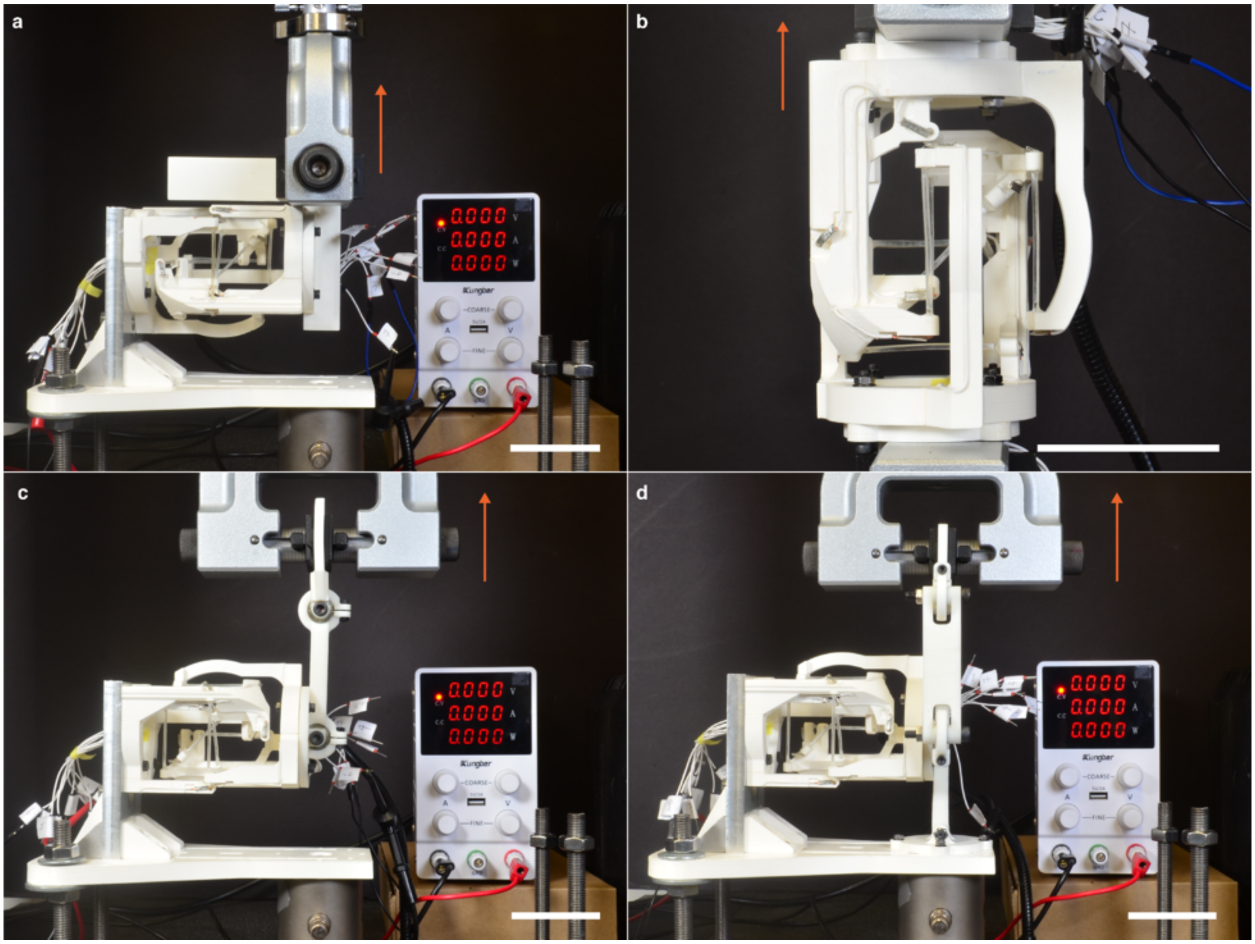

***Figure S39. Mechanical test setup of the 6-DOF device. a**, The test setup for the x- and y-translation-enabled modes. **b**, The test setup for the z-translation-enabled mode. **c**, The test setup for the x- and y-rotation-enabled modes. **d**, The test setup for the z-rotation-enabled mode. Scale bar, 50 mm.*

A wrist device was fabricated and tested three times under each mode along the two DOF (Fig. S40, Video S4). The loads were applied through a loading arc of radius 114.3 mm at a rate of 10 mm/min, corresponding to 5.01°/min. The displacement loads were determined based on the tested rotation axis and the device configuration mode. A displacement load of 20 mm (10.03°) was applied to the unlocked states. Conversely, the displacement load was lowered to 5 mm (2.50°) under the locked states to avoid damaging the device.

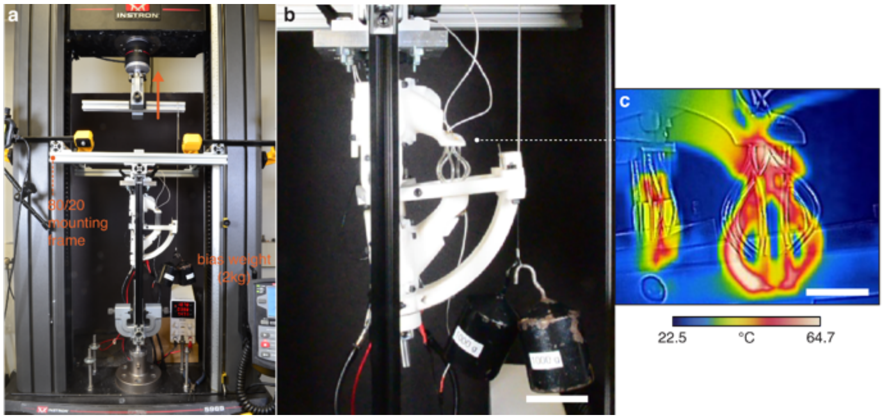

***Figure S40. Mechanical testing of the wrist joint isolated from the arm-wearable device. a**, The setup overview. **b**, The device at 20 mm displacement. Scale bar, 50 mm. **c**, A thermal image of the device at 20 mm displacement. Scale bar, 20 mm.*

Three thimble devices were tested for three cycles under each reconfiguration listed in Fig. 4h. The loads were applied (Fig. S41, Video S6) at 10 mm/min. The displacement loads varied between rod configuration modes. For configuration [0, 0, 0, 0, 0] where all rods remained stiff, a displacement load of 0.6 mm was used, which corresponded to a 3% system strain with respect to the bounding volume of the flexures (20 mm^3). Alternatively, the partially unlocked configuration [0, 0, 0, 0, 1] was subjected to a load of 3 mm (15% strain), whereas the rest of the modes were loaded with the designed maximum displacement of 5 mm (25% strain).

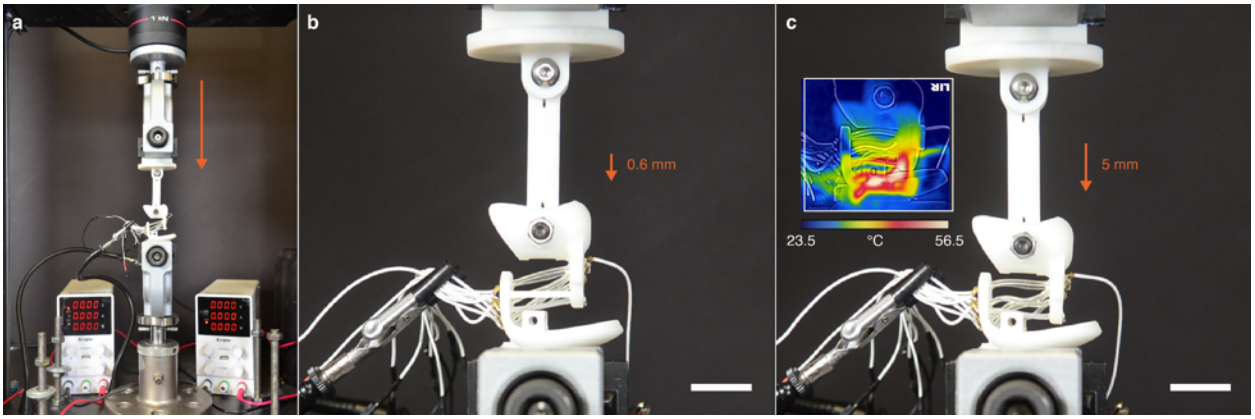

***Figure S41. Mechanical test setup of the haptic thimble device. a**, The setup overview. **b**, The device in the [0, 0, 0, 0, 0] configuration with a compressive displacement of 0.6 mm. **c**, The device in the [1, 1, 1, 1, 1] configuration with a compressive displacement of 5 mm. Scale bar, 20 mm.*

6.5 Mechanical Testing Jig Design

In the Instron tests, we designed the jigs without slide-contact joints. The parts are either clamped or fixed to the Instron machine or mechanically articulated. Each rotational axis annotated in figures S41 through 45 is implemented with a metallic bearing between moving parts to minimize friction and avoid confounding measurements.

The 6-DOF device was tested using customized jigs attached to the Instron machine for applying loads along the appropriate axes. The jigs contained two parts: a fixture that attached the device's fixed end to the Instron machine's fixed base (Fig. S42a) and a loading mechanism that applied loads. The jigs were 3D printed using either PLA or UV curable resin except for the mounting plate made with machined 6061 aluminum to minimize deflections when loading the devices (Fig. S42b). To test translations along the X and Y-axis, a rectangular plate was bolted to the device's free end and clamped to the load cell (Fig. S42d). Rotations about the X and Y axes were tested similarly, but rotational bearings and arms were added to the loading mechanism to accommodate the rotation (Fig. S42e). Translation along the Z-axis was tested by attaching and clamping on 3D-printed plates bolted to both ends of the device (Fig. S42c). Finally, rotation about the Z-axis was tested with the pure rotational moment. The device's free end was connected to the base fixture via a rotational bearing coaxial to the Z-axis. This design constrained all motions except for rotation resulting from the torque applied through a loading arm 50 mm away from the axis (Fig. S42f). Similar to the X and Y-axis rotations, additional bearings and arms were added to the loading mechanism to accommodate the device's rotation.

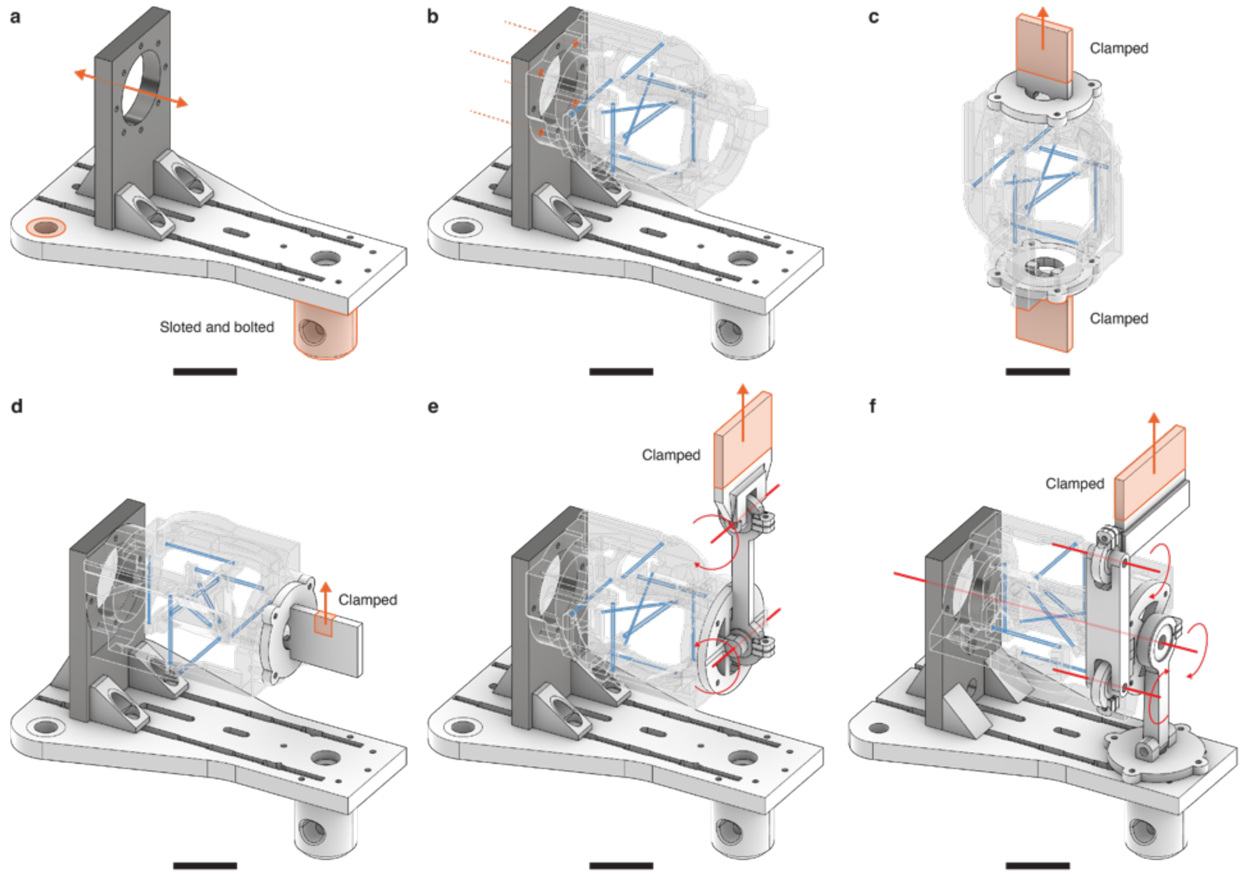

***Figure S42. The 6-DOF device test jig design. a**, The mounting plate and fixtures used to secure the device to the base of the intron machine. The mounting plate's position can be adjusted along the annotated axis. **b**, The device is bolted to the mounting plate for tests. **c**, the z-translation test jig design. **d**, The x-translation test jig design. Y-translation was tested with the same design with the device reoriented. **e**, The x-rotation test jig design. Y-rotation was tested with the same design with the device reoriented. **f**, The z-rotation test jig design. Scale bar, 50 mm.*

The wrist device was mounted on an articulated testing jig to simulate the human skeleton (Fig. S43a). An articulated U joint connected two stainless steel rods to simulate the two rotational DOF at the human wrist joint. The two stages were mounted on stainless steel rods with interfacing adaptors made with the Formlabs UV-curable resin (Fig. S43b). The articulated device and jig were then mounted to a machined aluminum stock and frame with flanged mounts and bolts (Fig. S43c). Secondly, the aluminum frame was clamped to the Instron machine to minimize shakes and undesired displacements through the test (Fig. S40a).

The free end had been added with loading arcs centered on the flexion and deviation rotation axes for applying displacement loads (Fig. S43d). The free end was connected to the load cell with a stainless-steel cable, and a bias weight of 2 kg was added to the arc in the opposite direction to ensure the cable remained straightened throughout the test. To test the device along a rotational

DOF, an additional bearing was added in parallel to the U-joint to constrain the device to displace about the axis of interest while eliminating unwanted displacements (Fig. S44, S45).

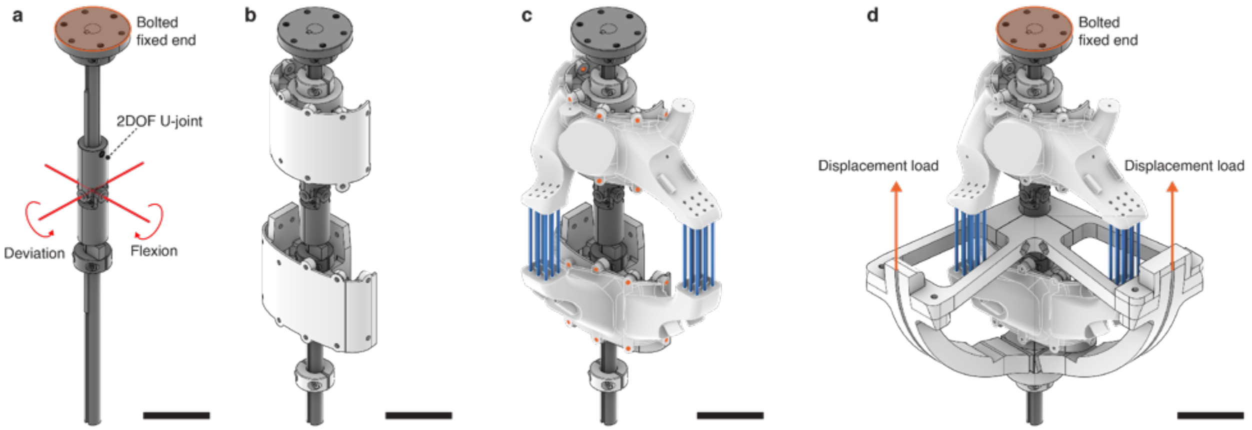

***Figure S43. The isolated wrist joint's test jig design. a**, the articulation assembled using metal parts. **b**, The 3D printed adapter parts to interface the jig with the device. **c**, The jig with the device mounted and secured through the bolt holes marked in orange. **d**, The loading arcs used to apply weight. Scale bar, 50 mm.*

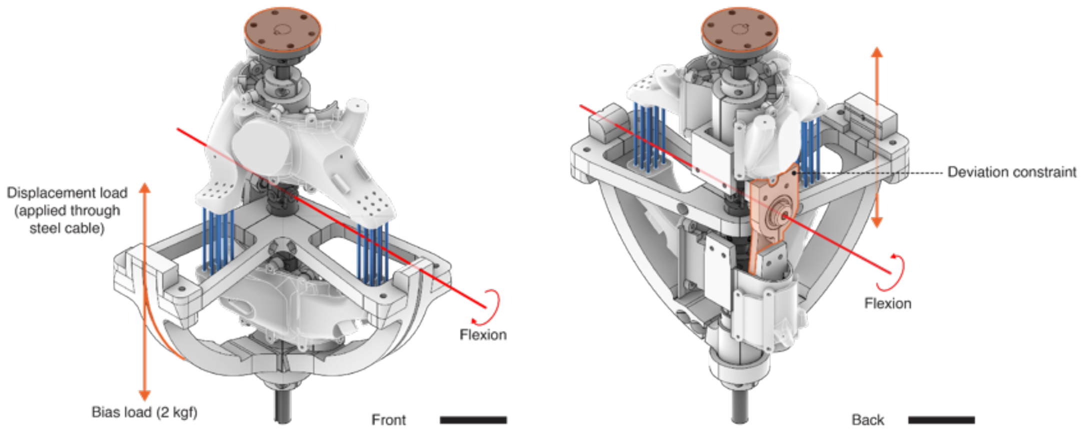

***Figure S44. Wrist joint test jig configuration for the flexion DOF.** Scale bar, 50 mm.*

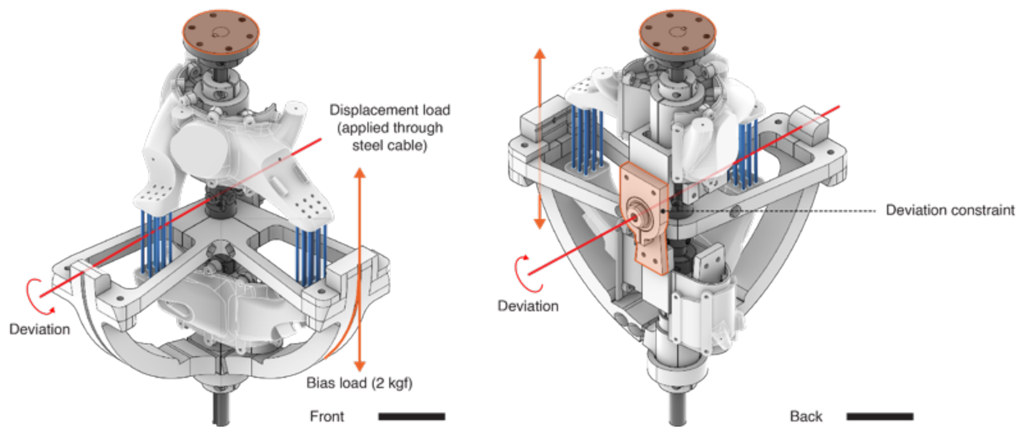

Figure S45. Wrist joint test jig configuration for the deviation DOF. *Scale bar, 50 mm.*

The thimble devices were modeled and fabricated with additional mechanical features on the two stages for mechanical tests (Fig. S46). The bottom stage was printed with a rectangular plate for clamping by the Instron machine on the fixed end. The displacement loads were applied to the free end at the fingertip contact area to simulate the designed use case. Two bearings and a linkage were added between the free end and the clamp to accommodate rotations during the load cycles.

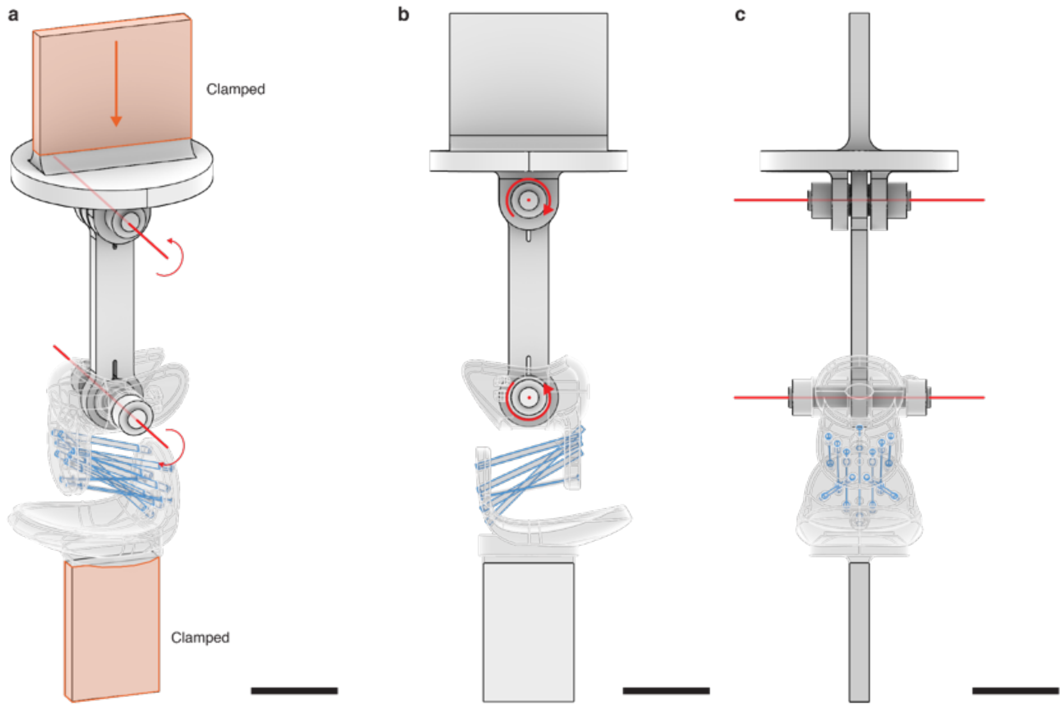

***Figure S46. The haptic thimble test jig design. a**, The test jig design and loading mechanism articulation to accommodate device displacements in the other directions. **b**, The device and jig, viewed at the front. **c**, The device and jig, viewed at the side. Scale bar, 20 mm.*

6.6 Device Repeatability

We conducted an additional repeatability test on the thimble device (Fig. S47), revealing that the device consistently performed over 100 loading-unloading cycles. The device was configured into three modes (fully locked [0, 0, 0, 0, 0], partially unlocked [0, 1, 0, 0, 1], and fully unlocked [1, 1, 1, 1, 1]) and displacement-loaded (0.6 mm, 5 mm, and 5mm, respectively) to measure their response across 100 cycles. The device was not heating/cooling-cycled between repetitions. For the fully locked state, the observed force remained constant at 30 N over 100 cycles (50 Nmm^{-1} in stiffness). Similarly, the forces remained constant at 1.1 N over 100 cycles when fully unlocked. The forces for the partially locked mode remained constant (20 N) for around 80 cycles and further decreased to 16 N for the remainder. We speculate that a potential reason for the decreased in force/stiffness is due to heat accumulation and diffusion from hot to cold flexures over time.

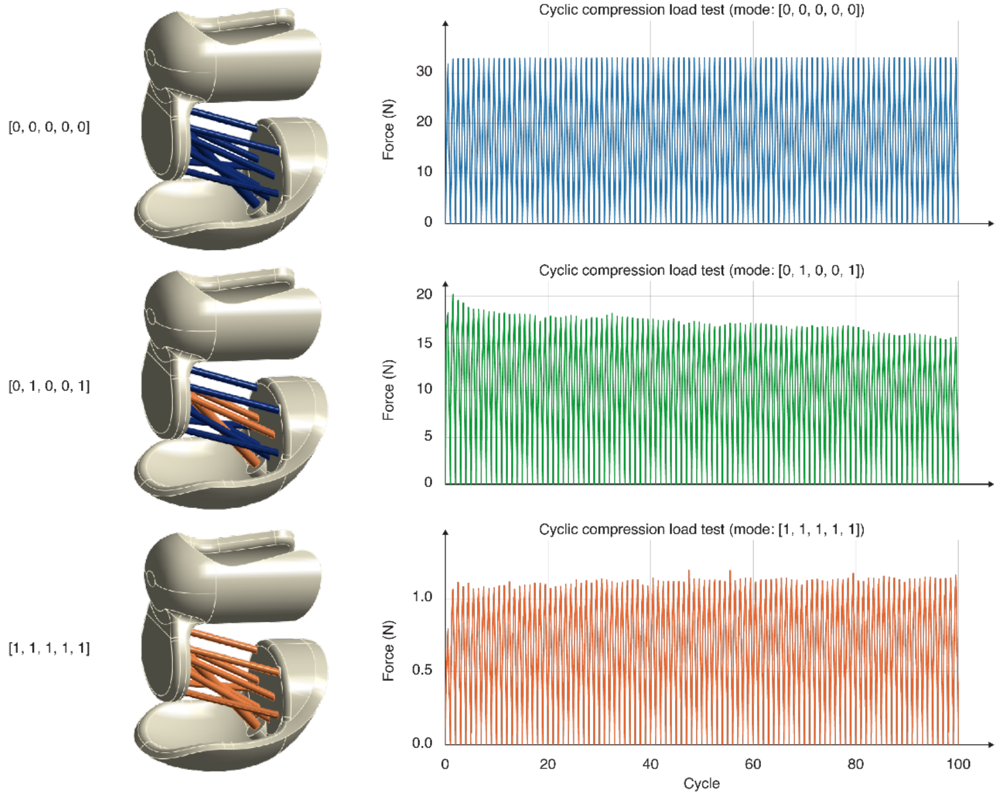

Figure S47. The haptic thimble repeatability test over 100 loading cycles.

On the other hand, we found no visible performance degradation when repeatedly heating and cooling a single rod (OD 2 mm). In this test, a rod was repeatedly cycled between RT and 54°C. In each cycle, the rod was heated or cooled for three minutes then compressed by 1% for four cycles to measure its response. Figure S48 shows the measurements of a single rod's mechanical response under 1% compression over ten heating and cooling cycles. There was no visible performance drop or trend in both heated and unheated states.

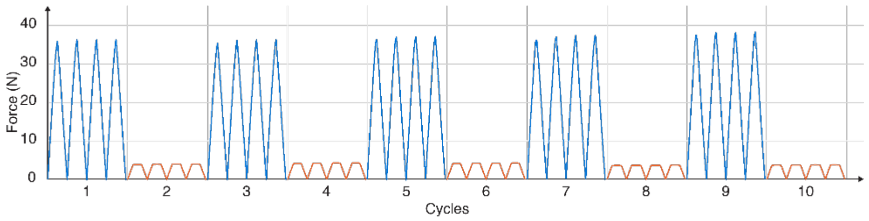

Figure S48. An OD 2 mm rod's compression test over ten heating/cooling cycles. *The rod is conditioned at ambient temperature at odd number cycles (blue) and 54℃ at even number cycles (orange).*

6.7 Literature Benchmark

The literature comparison and benchmark figure (Fig. 1b) was composed by analyzing the data and devices presented in prior research pertaining to stiffness-changing devices. For each work, the max programmable DOF was determined based on the most complex (i.e., possessing the most reconfigurable DOF) device that had been demonstrated. However, if a generalized design pipeline was presented (e.g., ReCompFig[15]), we then reported the highest number of achievable DOF using the pipeline. The stiffnesses under the softened and hardened modes were calculated at 1% displacement load with respect to device height. We used source data from mechanical load tests when it was available. However, in the case that the source data was not attainable, we used image processing and analysis software (ImageJ) to read out the values from the load curves presented in the data figures. Additionally, if a load curve was presented in different units (e.g., Stress over displacement or load over strain), we converted the measurements by factoring in the device dimensions to generate a systematic, effective stiffness readout in Nmm^{-1}.

On the other hand, the stiffness requirements for kinesthetic wearable device design could vary depending on the targeted joint, which has varying isometric strengths and just noticeable displacements. We use several extreme cases to illustrate the range of interest in a kinesthetic design context. The lower bound of 0.013 Nmm^{-1} was determined based on the stiffness JND of the index finger, which was one of the most sensitive receptors on the body and frequently used in haptic explorations[23]. On the other hand, the upper bound of 59.342 Nmm^{-1} was calculated by superimposing a wearable device design context to the data provided by Gupta et al.[20] and Ramos et al. [24]. The elbow joint has an isometric strength[20] of 72.5 Nm and a rotational proprioceptive JND [24] of 7 degrees. Assuming a device is placed 100 mm away from the elbow joint rotation axis and should resist the isometric strength below or at the JND displacement, then the device's stiffness should be $\frac{72.5\times1000}{\left(7\times\frac{\pi}{180}\right)\times100^2} = 59.342$ Nmm^{-1}.

Supplementary Table S6. Literature benchmark

Literature	Max programmable DOF	1% displacement stiffness (softened, Nmm^{-1})	1% displacement stiffness (hardened, Nmm^{-1})

Yang et al.[15]	5	0.970	10.204
Zappetti et al.[25]	2	0.577	9.813
Mueller et al.[26]	1	2.356	235.573
Liu et al.[27]	1	1.526	58.470
Mekaouche et al.[28]	1	0.196	0.247
Chen et al.[29]	1	0.020	0.383
Mintchev et al.[30]	3	0.103	2.410
Kuppens et al.[31]	1	0.305	9.631
Gao et al.[32]	1	0.182	5.099
Shimohara et al.[33]	2	0.561	5.689
Us (haptic thimble)	1	2.270	52.815
Us (6DOF-X_T)	6	8.176	20.161
Us (6DOF-Y_T)	6	6.856	27.807
Us (6DOF-Z_T)	6	16.692	73.785
Us (6DOF-X_B)	6	4.250	13.125
Us (6DOF-Y_B)	6	2.445	7.613

6.8 Camera and Thermal Images

Images and videos were recorded with Sony (A7 III) and Canon (5D Mark II) DSLR cameras. Thermal images were recorded using a thermal imaging camera (HTi, HT-19). All images and videos were used without post-processing except for adjusting brightness and contrast for readability. Videos were composed using Adobe Premiere Pro.

List of Supplementary Figures

Figure S1. Stiffness-changing flexural rod casting jig design.

Figure S2. The fabrication process of stiffness-changing flexural rods.

Figure S3. Structure assembly and connection.

Figure S4. Thermal images of the 6-DOF device under different mode configurations.

Figure S5. Screw algebraic representation of a single flexural rod.

Figure S6. Using screw linear subspaces to represent a compliant joint kinematics and flexure design.

Figure S7. Constraint subspace Boolean differences.

Figure S8. Algorithm for designing kinematically reconfigurable compliant metastructure joints.

Figure S9. A step-by-step design example of the kinematics design algorithm using the wrist joint as an example.

Figure S10. The flexural rod and motional axis parameterization used by the analytical stiffness model.

Figure S11. Stiffness and design parameter sweep using the analytical model, normalized by the material's elastic modulus.

Figure S12. 6-DOF device FEA setup and simulation result.

Figure S13. Arm-wearable device FEA setup and simulation result.

Figure S14. Haptic thimble FEA setup and simulation result.

Figure S15. The constraint subspace selection process shown in Venn diagram representations.

Figure S16. The 6-DOF device design.

Figure S17. Alternative 6-DOF device design.

Figure S18. Arm-wearable device kinematic mode specification and Venn diagram (1DOF rotation).

Figure S19. Arm-wearable device kinematic mode specification and Venn diagram (2DOF rotation).

Figure S20. The forearm joint's design iteration.

Figure S21. The final forearm joint design performance evaluation using FE simulation.

Figure S22. The finger joints' design iteration.

Figure S23. The final finger joints design performance evaluation using FE simulation.

Figure S24. The wrist joint's design iteration.

Figure S25. The final wrist joint design performance evaluation using FE simulation.

Figure S26. The arm-wearable device design.

Figure S27. Pictures of the arm-wearable device's forearm joint in the unlocked state.

Figure S28. Pictures of the arm-wearable device's forearm joint in different unlocked modes.

Figure S29. Pictures of the arm-wearable device's finger joints in different unlocked modes.

Figure S30. The thimble device's kinematic mode specification and Venn diagram.

Figure S31. The thimble device's design iteration.

Figure S32. The Haptic thimble device design.

Figure S33. DMA test result for the epoxy sample.

Figure S34. Mechanical properties of Epoxy under room temperature and 54℃.

Figure S35. Flexural rod characterization test setup and results.

Figure S36. Flexural rod tensile characterization.

Figure S37.Characteristic flexural rod hysteresis plots.

Figure S38. Flexure rod recovery after bending and buckling.

Figure S39. Mechanical test setup of the 6-DOF device.

Figure S40. Mechanical testing of the wrist joint isolated from the arm-wearable device.

Figure S41. Mechanical test setup of the haptic thimble device.

Figure S42. The 6-DOF device test jig design.

Figure S43. The isolated wrist joint's test jig design.

Figure S44. Wrist joint test jig configuration for the flexion DOF.

Figure S45. Wrist joint test jig configuration for the deviation DOF.

Figure S46. The haptic thimble test jig design.

Figure S47. The haptic thimble repeatability test over 100 loading cycles.

Figure S48. An OD 2 mm rod's compression test over ten heating/cooling cycles.

List of Supplementary Tables

Supplementary Table S1: List of notations used throughout this work.

Supplementary Table S2. FE simulation material definitions.

Supplementary Table S3. FE simulation boundary conditions.

Supplementary Table S4. 6-DOF device constraint subspace table.

Supplementary Table S5. The arm-wearable device design criteria.

Supplementary Table S6. Literature benchmark

References

1. Zhong, K. et al. EpoMemory: Multi-State Shape Memory for Programmable Morphing Interfaces. in Proceedings of the 2023 CHI Conference on Human Factors in Computing Systems (Association for Computing Machinery, New York, NY, USA, 2023). doi:10.1145/3544548.3580638.
2. Engelberg, P. I. & Tesoro, G. C. Mechanical and thermal properties of epoxy resins with reversible crosslinks. Polym Eng Sci 30, 303–307 (1990).
3. Buckner, T. L., Bilodeau, R. A., Kim, S. Y. & Kramer-Bottiglio, R. Roboticizing fabric by integrating functional fibers. Proceedings of the National Academy of Sciences 117, 25360–25369 (2020).
4. Wienert, V., Sick, H. & zur Mühlen, J. Local thermal stress tolerance of human skin. Anasth Intensivther Notfallmed 18, 88–90 (1983).
5. Jeschke, M. G. et al. Burn injury. Nat Rev Dis Primers 6, 11 (2020).
6. Hopkins, J. B. & Culpepper, M. L. Synthesis of multi-degree of freedom, parallel flexure system concepts via Freedom and Constraint Topology (FACT) - Part I: Principles. Precis Eng 34, 259–270 (2010).
7. Hopkins, J. B. & Culpepper, M. L. Synthesis of multi-degree of freedom, parallel flexure system concepts via freedom and constraint topology (FACT). Part II: Practice. Precis Eng 34, 271–278 (2010).
8. Sun, F. & Hopkins, J. B. Mobility and Constraint Analysis of Interconnected Hybrid Flexure Systems Via Screw Algebra and Graph Theory. J Mech Robot 9, (2017).
9. Hopkins, J. B. & Culpepper, M. L. Synthesis of precision serial flexure systems using freedom and constraint topologies (FACT). Precis Eng 35, 638–649 (2011).
10. Shaw, L. A. et al. Computationally efficient design of directionally compliant metamaterials. Nat Commun 10, 291 (2019).
11. Hopkins, J. B. Designing hybrid flexure systems and elements using Freedom and Constraint Topologies. Mechanical Sciences 4, 319–331 (2013).
12. Hopkins, J. B. & McCalib, D. Synthesizing multi-axis flexure systems with decoupled actuators. Precis Eng 46, 206–220 (2016).
13. Hopkins, J. B., Vericella, J. J. & Harvey, C. D. Modeling and generating parallel flexure elements. Precis Eng 38, 525–537 (2014).
14. Lipkin, H. & Duffy, J. The Elliptic Polarity of Screws. Journal of Mechanisms, Transmissions, and Automation in Design 107, 377–386 (1985).
15. Yang, H. et al. ReCompFig: Designing Dynamically Reconfigurable Kinematic Devices Using Compliant Mechanisms and Tensioning Cables. in Proceedings of the 2022 CHI Conference on Human Factors in Computing Systems (Association for Computing Machinery, New York, NY, USA, 2022). doi:10.1145/3491102.3502065.
16. Su, H.-J., Shi, H. & Yu, J. A Symbolic Formulation for Analytical Compliance Analysis and Synthesis of Flexure Mechanisms. Journal of Mechanical Design 134, (2012).

17. Holst, G. L., Teichert, G. H. & Jensen, B. D. Modeling and Experiments of Buckling Modes and Deflection of Fixed-Guided Beams in Compliant Mechanisms. Journal of Mechanical Design 133, (2011).
18. Milner, T. E. & Franklin, D. W. Characterization of multijoint finger stiffness: dependence on finger posture and force direction. IEEE Trans Biomed Eng 45, 1363–1375 (1998).
19. Reissner, L. et al. Minimal detectable difference of the finger and wrist range of motion: comparison of goniometry and 3D motion analysis. J Orthop Surg Res 14, 173 (2019).
20. Gupta, A. & O'Malley, M. K. Design of a haptic arm exoskeleton for training and rehabilitation. IEEE/ASME Transactions on Mechatronics 11, 280–289 (2006).
21. Matsuoka, J., Berger, R. A., Berglund, L. J. & An, K.-N. An Analysis of Symmetry of Torque Strength of the Forearm Under Resisted Forearm Rotation in Normal Subjects. Journal of Hand Surgery 31, 801–805 (2006).
22. Vanswearingen, J. M. Measuring Wrist Muscle Strength. Journal of Orthopaedic & Sports Physical Therapy 4, 217–228 (1983).
23. Zoeller, A. C. & Drewing, K. A Systematic Comparison of Perceptual Performance in Softness Discrimination with Different Fingers. Atten Percept Psychophys 82, 3696–3709 (2020).
24. Ramos, M. M., Carnaz, L., Mattiello, S. M., Karduna, A. R. & Zanca, G. G. Shoulder and elbow joint position sense assessment using a mobile app in subjects with and without shoulder pain - between-days reliability. Physical Therapy in Sport 37, 157–163 (2019).
25. Zappetti, D., Jeong, S. H., Shintake, J. & Floreano, D. Phase Changing Materials-Based Variable-Stiffness Tensegrity Structures. Soft Robot 7, 362–369 (2019).
26. Mueller, J., Lewis, J. A. & Bertoldi, K. Architected Multimaterial Lattices with Thermally Programmable Mechanical Response. Adv Funct Mater 32, 2105128 (2022).
27. Liu, K. et al. 4D printed zero Poisson's ratio metamaterial with switching function of mechanical and vibration isolation performance. Mater Des 196, 109153 (2020).
28. Mekaouche, A., Chapelle, F. & Balandraud, X. A compliant mechanism with variable stiffness achieved by rotary actuators and shape-memory alloy. Meccanica 53, 2555–2571 (2018).
29. Chen, T., Pauly, M. & Reis, P. M. A reprogrammable mechanical metamaterial with stable memory. Nature 589, 386–390 (2021).
30. Mintchev, S., Salerno, M., Cherpillod, A., Scaduto, S. & Paik, J. A portable three-degrees-of-freedom force feedback origami robot for human–robot interactions. Nat Mach Intell 1, 584–593 (2019).
31. Kuppens, P. R., Bessa, M. A., Herder, J. L. & Hopkins, J. B. Compliant Mechanisms That Use Static Balancing to Achieve Dramatically Different States of Stiffness. J Mech Robot 13, (2021).
32. Gao, Y., Huang, X., Mann, I. S. & Su, H.-J. A Novel Variable Stiffness Compliant Robotic Gripper Based on Layer Jamming. J Mech Robot 12, (2020).

33. Shimohara, S., Lee, R. H. & Hopkins, J. B. Compliant mechanisms that achieve binary stiffness along multiple degrees of freedom. J Compos Mater 57, 645–657 (2022).
