# Supplementary material for: A compliant metastructure design with reconfigurability up to six degrees of freedom
Source: Nat Commun. 2025 Jan 16;16:719. doi: 10.1038/s41467-024-55591-2 (PMC11739503; doi:10.1038/s41467-024-55591-2)
Supplement: Supplementary file 2 — Description of Additional Supplementary Files [file 41467_2024_55591_MOESM2_ESM.pdf]

Description of Additional Supplementary Files

File Name: Video_S1

Description: 6DOF device. A video of the device configured under different DOF modes and moved by hand. Both real and thermal images are included.

File Name: Video_S2

Description: Individual joints of the arm-wearable device. A video of the wearable device worn by a user with close-up clips showing each joint under their enabled modes.

File Name: Video_S3

Description: Arm-wearable device fully enabled. A video of the wearable device with all joint mobilities enabled, worn by a user, and exercised along every DOF at the same time.

File Name: Video_S4

Description: Mechanical test of the arm-wearable device wrist joint. A video showing the mechanical test setup and process. The wearable device wrist joint was configured to enable flexion and loaded accordingly.

File Name: Video_S5

Description: Haptic thimble. Videos showing the haptic thimble configured to the fully rigid ([0, 0, 0, 0, 0]), partially softened ([0, 1, 0, 0, 1]), and fully softened modes ([1, 1, 1, 1, 1]). The image displayed on the surface shows the materials (aluminum, PU foam, jelly) the device was aimed to simulate given perceptual limits.

File Name: Video_S6

Description: Mechanical test of the haptic thimble device. A video showing the mechanical test setup and process. The device was configured under the fully softened mode ([1, 1, 1, 1, 1]).
